# Clock gene homologs* lin-42* and *kin-20* regulate circadian rhythms in *C. elegans*

**DOI:** 10.1038/s41598-024-62303-9

**Published:** 2024-06-05

**Authors:** Melisa L. Lamberti, Rebecca K. Spangler, Victoria Cerdeira, Myriam Ares, Lise Rivollet, Guinevere E. Ashley, Andrea Ramos Coronado, Sarvind Tripathi, Ignacio Spiousas, Jordan D. Ward, Carrie L. Partch, Claire Y. Bénard, M. Eugenia Goya, Diego A. Golombek

**Affiliations:** 1 Laboratorio de Cronobiología, Universidad Nacional de Quilmes, Buenos Aires, Argentina; 2 Department of Chemistry and Biochemistry, University of California Santa Cruz, Santa Cruz, USA; 3 Department of Biological Sciences, Université du Québec à Montréal, CERMO-FC Research Center, Montréal, QC Canada; 4 Department of Molecular, Cell & Developmental Biology, University of California Santa Cruz, Santa Cruz, USA; 5 Laboratorio Interdisciplinario del Tiempo (LITERA), Universidad de San Andrés/CONICET, Buenos Aires, Argentina; 6 Center for Circadian Biology, UC San Diego, La Jolla, CA USA; 7 Department of Neurobiology, University of Massachusetts Chan Medical School, Worcester, MA USA; 8 European Institute for the Biology of Aging, University Medical Center Groningen, Groningen, The Netherlands

**Keywords:** Circadian mechanisms, Circadian regulation

## Abstract

Circadian rhythms are endogenous oscillations in nearly all organisms, from prokaryotes to humans, allowing them to adapt to cyclical environments for close to 24 h. Circadian rhythms are regulated by a central clock, based on a transcription-translation feedback loop. One important protein in the central loop in metazoan clocks is PERIOD, which is regulated in part by Casein kinase 1ε/δ (CK1ε/δ) phosphorylation. In the nematode *Caenorhabditis elegans*, *period* and *casein kinase 1ε/δ* are conserved as *lin-42* and *kin-20*, respectively. Here, we studied the involvement of *lin-42* and *kin-20* in the circadian rhythms of the adult nematode using a bioluminescence-based circadian transcriptional reporter. We show that mutations of *lin-42* and *kin-20* generate a significantly longer endogenous period, suggesting a role for both genes in the nematode circadian clock, as in other organisms. These phenotypes can be partially rescued by overexpression of either gene under their native promoter. Both proteins are expressed in neurons and epidermal seam cells, as well as in other cells. Depletion of LIN-42 and KIN-20, specifically in neuronal cells after development, was sufficient to lengthen the period of oscillating *sur-5* expression. Therefore, we conclude that LIN-42 and KIN-20 are critical regulators of the adult nematode circadian clock through neuronal cells.

## Introduction

Circadian rhythms are endogenous oscillations that allow adaptation to our cyclic environment, maintaining an intrinsic periodicity close to 24 h. Maintained under constant conditions as free running rhythms (FR), circadian rhythms are synchronized by environmental signals (‘time givers’, or zeitgebers) such as light and temperature, and their period remains relatively unchanged by variations in temperature^1^. In metazoans, circadian rhythms are modulated by central and peripheral clocks that depend on the activity of a group of evolutionarily conserved clock genes that regulate interlocked transcription-translation feedback loops (TTFL)^2^. In mammals, CLOCK and BMAL1 proteins form a heterodimer that acts as a positive element in the core feedback loop, activating the transcription of clock genes, such as PERIOD (PER) and CRYPTOCROME (CRY), by binding to the E-boxes in their promoters. In turn, PER binds to CRY and CK1ε/δ, forming multimeric complexes that function as the negative element in the feedback loop by inhibiting the activity of CLOCK:BMAL1, thereby hindering their own transcription and closing the TTFL^3–5^. PER proteins are progressively phosphorylated by CK1ε/δ to regulate their stability, resulting in daily changes in PER levels and CLOCK/BMAL1 activity^6^.

Circadian rhythms of physiological and behavioral variables, such as gene expression and locomotor activity, have been observed in the nematode *C. elegans*^7^; however, the molecular mechanism of its central pacemaker is currently unknown. With the exception of *cry* (*cryptochrome*), *C. elegans* has homologs to other metazoan clock genes such as *lin-42* (*period*), *kin-20* (CK1ε/δ), *aha-1* (*cycle/bmal1*), *nhr-23* (*rorɑ/ɣ*), *nhr-85* (*rev-erbɑ/β*), and *nhr-25* (*sf-1*)^8–11^. Some of these genes are well characterized and play other functions in the nematode, like the regulation of developmental timing^12–14^, and they have been proposed to have evolved to be exclusively part of a developmental clock. However, their function in circadian rhythms in adults has been understudied at the molecular level. Remarkably, *nhr-23* (*rorɑ/ɣ*), which was previously shown to be essential for the molting clock^15^, was very recently found also to be essential for circadian transcriptional rhythms in adult nematodes^16^, as previously shown in mammals. This supports the idea that the ancestral circadian clock genes might have evolved in *C. elegans* to function over multiple periods depending on the biological context.

LIN-42 has seven different isoforms (isoforms a–g) that are relevant in the development of the nematode to different extents. For example, the absence of *lin-42a* (allele *ok2385*) or *lin-42b* (allele *ox461*) generates larval arrest and developmental defects^12^. By contrast, the absence of *lin-42c* (allele *n1089*) generates a less severe phenotype^17^ and little is known about the other isoforms. *lin-42* is expressed in epidermal cells, seam cells (multipotent lateral epidermal cells that undergo multiple divisions during larval development), the pharynx, and neuronal cells^12,18,19^. In the complete absence of *lin-42*, epidermal seam cells, vulval precursor cells, and the migration of sex myoblasts all develop precociously^19^. *lin-42* mRNA levels oscillate during each of the four molting cycles of postembryonic development (approximately 8–10 h at 25 °C)^18^, regulating developmental timing and entrance into an alternative dauer larval stage^12,17,19^, likely through gene expression modulation^20–23^.

The *kin-20* gene encodes seven isoforms (isoforms a–g) and is expressed in seam cells and neurons^8^. Dysfunctional KIN-20 proteins cause developmental and molting defects^8,24^. KIN-20 also regulates the expression of *lin-42* in the larval stages^23^. In adult nematodes, KIN-20 stabilizes nervous system architecture after axon outgrowth^24^.

Previous studies suggested that LIN-42 and KIN-20 are involved in nematode circadian rhythms. *lin-42* loss-of-function mutants show defects in circadian rhythms of locomotor activity^25^, as is the case for PER mutants in other metazoa^26,27^. Likewise, pharmacological inhibition of KIN-20 activity with the CKIδ/ε-selective kinase inhibitor PF-670462 significantly lengthens the period of *sur-5* bioluminescence rhythms in nematodes^28^, similar to its effects on clocks in diverse species, including plants, *Drosophila* and mammals^29–31^. However, the functional roles of LIN-42 and KIN-20 in *C. elegans* adult circadian rhythms have not been deeply characterized at the molecular level. This is due, in part, to the complexity of the different isoforms from both genes and the lack of tools to investigate the functional role of these proteins in adult circadian rhythms. We hypothesized that LIN-42 and KIN-20 play an important role in regulating circadian rhythms in *C. elegans*, similar to what occurs in other models such as mammals and *Drosophila*. We tackled this by using complementary approaches, combining biochemistry with genetic manipulations targeting the different isoforms of LIN-42 and KIN-20, and protein degradation post-development.

In this study, we characterized the effects of *lin-42* and *kin-20* disruption on circadian rhythms in adult nematodes utilizing a sensitive bioluminescence-based reporter system^28^. We observed significant changes in the luminescent circadian rhythms driven by the *sur-5* promoter in *lin42(ox461)*, *kin-20(ok505)*, and *kin-20(ox463)* mutants. After development, the joint neuronal depletion of both LIN-42 and KIN-20 proteins, using an auxin-inducible degradation (AID) system, altered the transcriptional rhythms of *sur-5*. This effect was absent, however, when the proteins were depleted in seam cells, highlighting the specificity of these genes in neurons to regulate circadian rhythms. We also show that the quaternary architecture of LIN-42’s structure is conserved with mammalian PER2, suggesting that LIN-42 and KIN-20 may retain some functional similarity in the regulation of the circadian clock of adult nematodes.

## Results

### The quaternary architecture of LIN-42 is structurally similar to mouse PER2

LIN-42 has long been established as a homolog to PER^18^, which has essential roles in metazoan circadian rhythms. While *M. musculus* and *D. melanogaster* PER have a similar function as transcriptional co-repressors in the TTFL, the molecular details of the *Drosophila* TTFL differ from most other metazoans, which are genetically wired more similar to mammalian clocks^32^. Mammalian PER proteins are largely intrinsically disordered but contain four domains necessary for their circadian function: two tandem PAS domains (Period-Arnt-Sim, PAS-A, and PAS-B) that homodimerize PER, a CK1 binding domain (CK1BD), and a CRY-binding domain (CBD). The longest isoform of LIN-42 is approximately half the length of mouse PER2 (598 vs. 1225 amino acids), and it has conserved PAS and CK1 binding domains^19^ (Fig. 2A).

**Figure 1:**
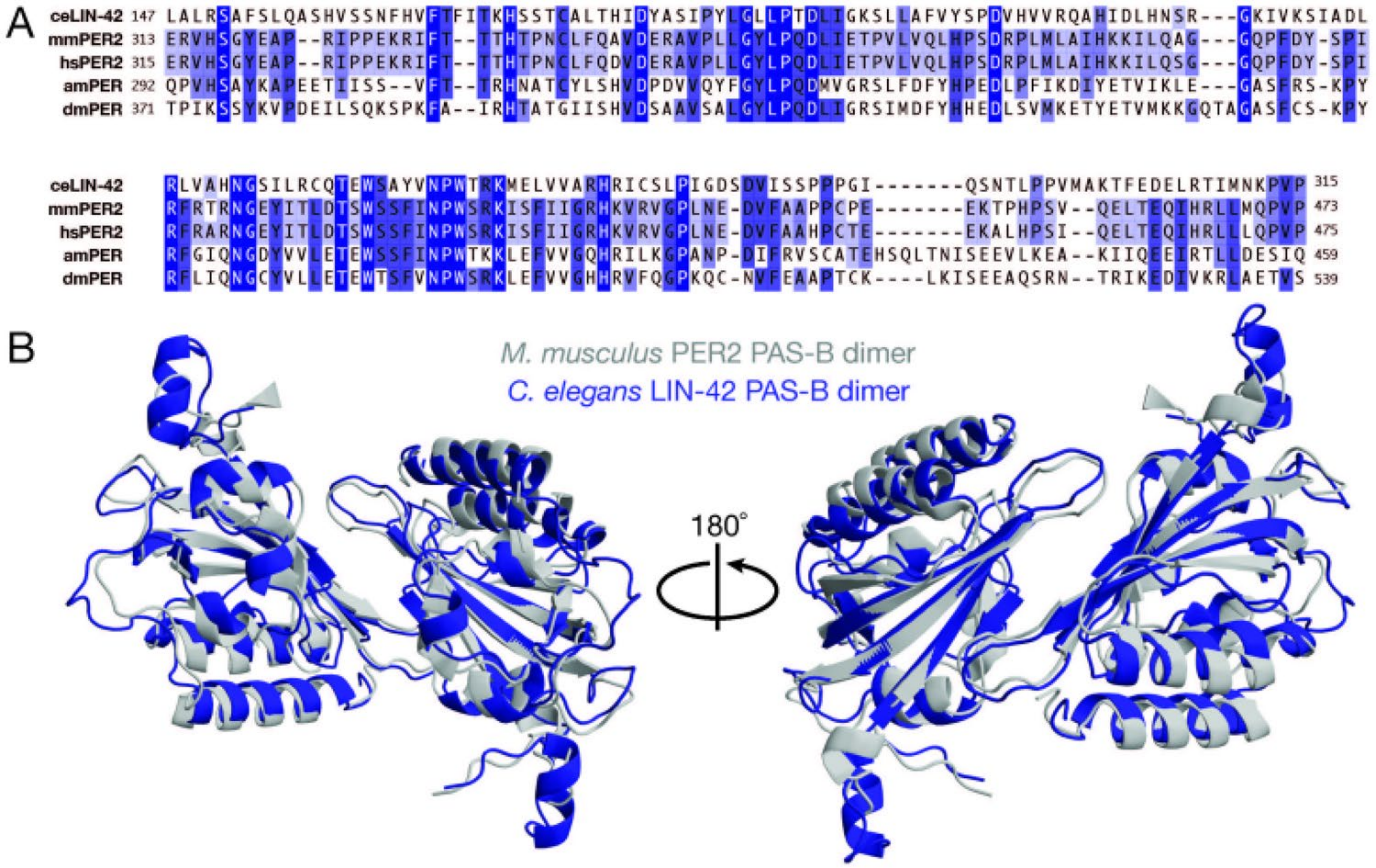
Conservation of mammalian PER quaternary structure in *C. elegans* LIN-42. (**A**) Sequence alignment of *C. elegans, M. musculus, H. sapiens, A. mellifera,* and *D. melanogaster* PER PAS-B domains. (**B**) Structural alignment of *C. elegans* LIN-42 PAS-B dimer (PDB 8GCI, blue) and *M. musculus* PAS-B dimer (PDB 3GDI, gray).

The PER proteins from *Drosophila* and mice exhibit different modes of dimerization by their PAS-B domains^33–35^, which may influence their function at the molecular level. The LIN-42 PAS-B domain is 24.4% and 24.0% identical to mouse PER2 and *Drosophila* PER, respectively (Fig. 1A). To gain insight into the function of the *C. elegans* PAS domains, we determined the structure of the LIN-42 dimer. Although we crystallized the protease-resistant core of the LIN-42 N-terminus (Fig. S1A,B), density was poor for the putative N-terminal PAS domain (residues 41–146), which deviates from ideal PAS domain topology^36^ (Fig. S1C). However, we were able to build a model of the LIN-42 PAS-B dimer resolved to 2.4 Å (Fig. 1A, Supplementary Table 1) that closely resembles the mouse PER2 PAS-B dimer (Fig. 1B). This was similar to the structural prediction by AlphaFold, which had two tandem PAS domains at high confidence, followed by intrinsic disorder (Fig. S1D,E). Altogether, this demonstrates a conserved dimerization strategy of LIN-42/PER among worms and mammals that may also manifest in functional similarities.

### LIN-42 modulates period length and circadian entrainment in adult nematodes

**Figure 2:**
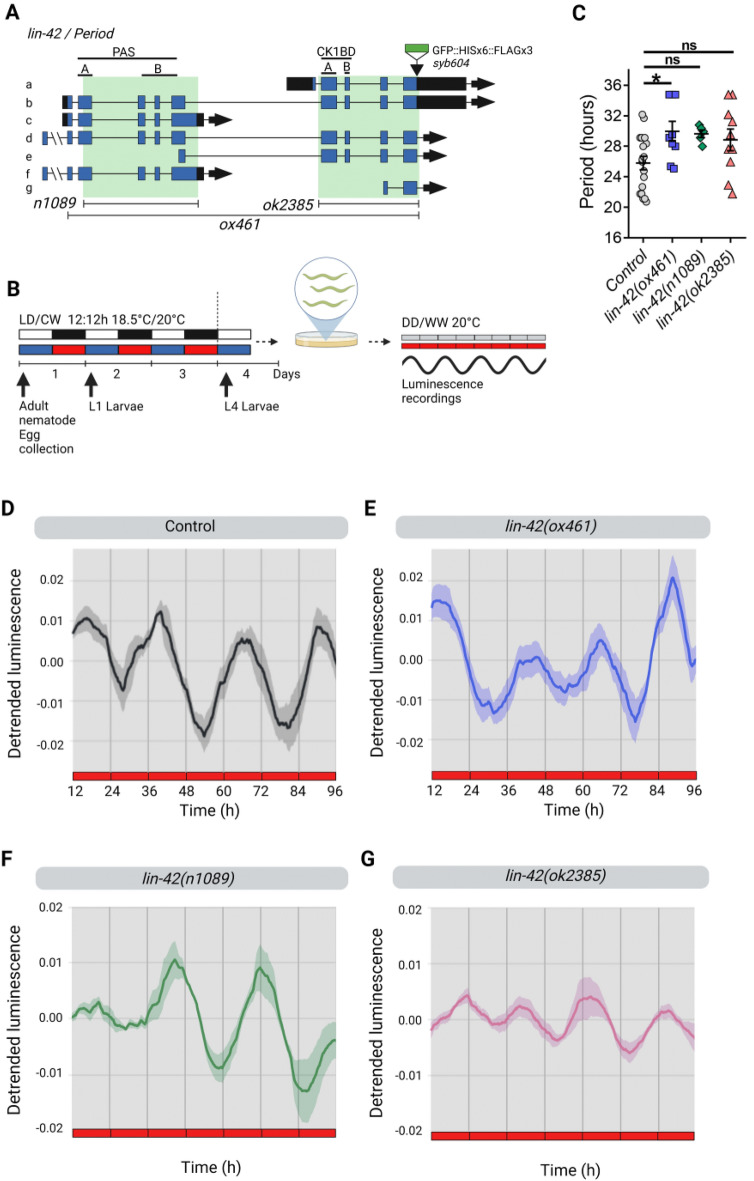
LIN-42 modulates period length under constant conditions. (**A**) The *C. elegans lin-42* locus. The *n1089* allele is a deletion of the PAS-A and B domains, the *ok2385* allele is a mutation that affects the CK1BD (Casein Kinase 1 Binding Domain), and the *ox461* allele is a complete deletion that lacks both (PAS and CK1BD). (**B**) Schematic assay of the luminescence experiments and photo/thermoperiodic conditions. Black/white bars indicate dark/light; blue/red bars indicate cold/warm; gray and red bars indicate the FR conditions. Nematodes were grown under dual cyclic conditions of 12 h:12 h Light/Dark (LD, ~ 150/0 µmol/m^2^/s) and Cold/Warm (CW, 18.5 °C/20 °C); ZT0, lights on and cold temperature phase onset, on NGM plates with a lawn of *E. coli* HB101. Embryos were collected from adult nematodes, and then L1 (larval stage 1) nematodes were grown on NGM plates with bacteria for 3 days. Once at the L4 stage, approximately 50 nematodes were transferred to the liquid luminescence media. For seven days, luminescence assays were performed under FR conditions (DD/WW, 20 °C). (**C**) Average endogenous period was determined for hours 18 h to 35 h for nematodes exhibiting a rhythm in FR. Error bars represent SEM. The average endogenous period of mutant strains of *lin-42* was compared with the control strain (25.79 ± 0.88 h, n = 18): *ox461* mutant (29.96 ± 1.30 h, n = 9), *n1089* mutant (29.63 ± 0.49 h, n = 5) and *ok2385* mutant (28.83 ± 1.43 h, n = 10), *p = 0.0299; ns, p > 0.05, one-way ANOVA followed by Dunnett´s multiple comparisons test. The analysis includes three biological replicates for each strain. Average reporter activity of rhythmic adult populations under FR conditions (DD, 20 °C), control (**D**) and *lin-42(ox461)* (**E**), *lin-42(n1089)* (**F**), *lin-42(ok2385)* (**G**). Luminescence signals are shown as mean ± SEM. The average reported activity was displayed with populations showing a similar first peak, and representative single traces are shown in Fig. S2. Each population consisted of 50 adult nematodes per well.

To probe the functional role of LIN-42 in the *C. elegans* circadian system, we used the luciferase reporter *psur-5::luc::gfp* as a clock output^28^, integrating the construct into the nematode genome by UV radiation (control strain *qvls*8, Supplementary Table 2). We then introduced the reporter into *lin-42* mutant backgrounds by genetic crosses with this control strain. First, we tested three different mutants for LIN-42 (Fig. 2A), each of which has deleted domains important for mammalian PER functions (Supplementary Table 2). *lin-42(ox461)* is a null allele with a 10,226 bp deletion lacking the N-terminal PAS-A and B domains and the C-terminal CK1BD^5,37–39^. The CKBD possesses two conserved motifs previously referred to as the SYQ and LT domains by Edelman et al.^19^, now termed CK1BD-A and CK1BD-B^37^. We also tested the effects of *lin-42(n1089)*, which has a deletion of 5233 bps, affecting only the PAS domains, and *lin-42(ok2385)*, which has a 2632 bp deletion, missing the CK1BD domain and downstream C-terminus.

Animals were synchronized to a dual LD/CW cycle (~ 150/0 µmol/m^2^/s; 18.5 °C/20 °C), and the bioluminescent activity of the *sur-5* promoter was measured thereafter in adult nematodes in constant conditions of darkness and 20 °C (DD/WW) as described in Goya et al.^28^ (Fig. 2B). To minimize behavioral variability due to developmental defects, L4-stage animals were manually selected for all the experiments. Rhythmic *sur-5* promoter activity occurred in all strains under constant conditions, indicating that the nematode exhibits free-running circadian rhythms (FR) (Fig. 2D–G and Fig. S2A–I). Although there was considerable high variability of circadian period across the mutant populations, we found that *lin-42(ox461)* animals showed a significantly longer period than the control strain (29.96 ± 1.30 h, n = 9 mutant rhythmic, n total = 24, 38% rhythmic vs. 25.79 ± 0.88 h, n = 18 control rhythmic, n total = 28, 64% rhythmic) (Fig. 2C–E and Fig. S2B). However, no significant difference was observed in the mutants of the PAS domains (Fig. 2C,F, and Fig. S2C, 29.63 ± 0.49 h, n = 5 mutant rhythmic, n total = 12, 42% rhythmic vs. control) and the CK1BD domain (Fig. 2C,G, and Fig. S2D, 28.83 ± 1.42 h, n = 10 mutant rhythmic, n total = 16, 63% rhythmic vs. control). Given that an abnormal circadian phenotype manifests only when both PAS and CK1BD domains are absent, this suggests that either is sufficient for circadian rhythm regulation.

**Figure 3:**
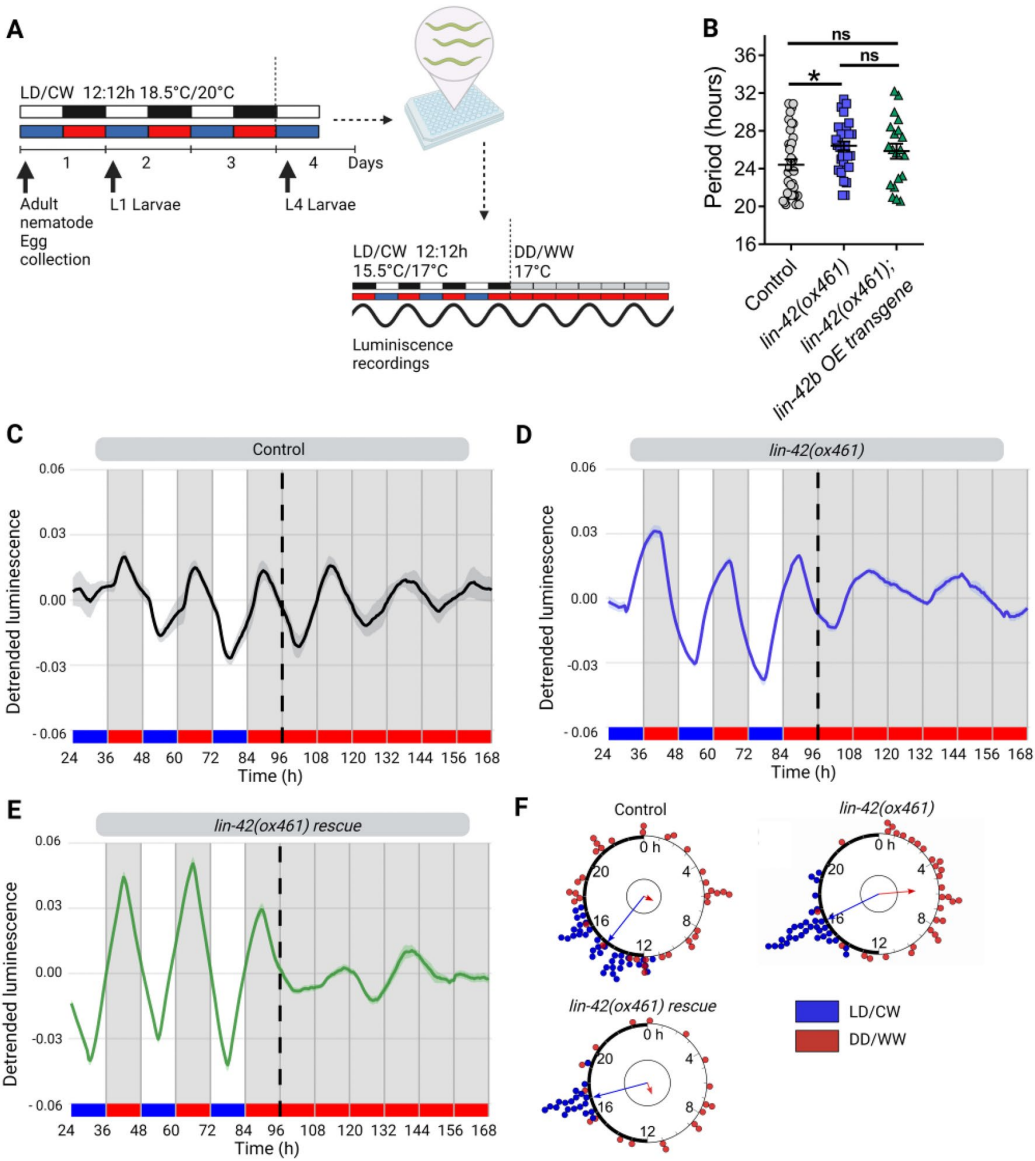
LIN-42 modulates circadian entrainment in adult nematodes. (**A**) Schematic assay of the luminescence experiments and photo/thermoperiodic conditions. Black/white bars indicate dark/light; blue/red bars indicate cold/warm; grey and red bars indicate the FR conditions. Nematodes were grown under dual cyclic conditions of 12 h:12 h Light/Dark (LD, ~ 150/0 µmol/m^2^/s) and Cold/Warm (CW, 18.5 °C/20 °C); ZT0, lights on and cold temperature phase onset, on NGM plates with a lawn of *E. coli* HB101. Embryos were collected from adult nematodes, hatched in the absence of food, and staged L1 (larval stage 1) nematodes were grown on NGM plates with bacteria for three days. Once at the L4 stage, approximately 50 nematodes were transferred to the liquid luminescence media. Luminescence assays were performed under dual cyclic conditions 12 h: 12 h of Light/Dark (LD, ~ 150/0 µmol/m^2^/s) and Cold/Warm (CW, 15.5 °C/17 °C) for three days; ZT0, lights on and cold temperature phase onset. Then, the luminescence was measured for four more days under FR conditions (DD/WW, 17 °C). (**B**) Average endogenous period of rhythmic populations of control (24.4 ± 0.57 h, n = 37), *ox461* mutants (26.41 ± 0.47 h, n = 32), and *lin-42b* overexpression (OE) transgene strain (25.85 ± 0.79 h, n = 20). One-way ANOVA followed by Tukey's multiple comparisons test, *p = 0.0302; ns, p > 0.05. Representative luciferase activity rhythms of adult populations under dual cyclic conditions (LD/CW, ~ 150/0 µmol/m^2^/s; 15.5 °C/17 °C) and FR conditions (DD, 17 °C), control (**C**), *lin-42(ox461)* (**D**) and *lin-42b* transgene (**E**). Luminescence signals are shown as mean ± SEM. The average reported activity was displayed with rhythmic populations showing a similar first peak in FR. Each population consisted of 50 adult nematodes per well, and the analysis included three biological replicates for each strain. The reported activity from all the wells for each strain is displayed in Fig. S3. (**F**) Rayleigh plots showing the phase of the bioluminescent peak under dual cyclic conditions (LD/CW, blue dots) and the first bioluminescent peak on the first day of release to FR (DD/WW, red dots) for rhythmic population nematodes: Control (LD/CW: 14.55 ± 0.27 h, n = 37; R = 0.90 and DD/WW: 7.85 ± 0.85 h, n = 37; R = 0.07), *lin-42(ox461)* mutants (LD/CW: 16.31 ± 0.20 h, n = 32; R = 0.95 and DD/WW: 5.68 ± 0.66 h, n = 32; R = 0.51) and *lin-42(ox461);lin-42b* OE transgene rescue (LD/CW: 17.03 ± 0.15 h, n = 20; R = 0.98 and DD/WW: 10.75 ± 1.09 h, n = 20; R = 0.17). Arrows represent each group’s average peak phase of *sur-5::luc* expression (mean vectors for the circular distributions). The length of the vector represents the strength of the phase clustering, while the vector’s angle represents the mean phase. Individual data points are plotted outside the circle. The central circle represents the threshold for p = 0.05.

To further characterize the effect of the *lin-42* full deletion on clock synchronization, we analyzed luminescence rhythms first under cyclic and then under constant conditions. We measured the luminescence of adult nematode populations over three days in LD/CW cycles (~ 150/0 µmol/m^2^/s; 15.5 °C/17 °C) and then for four days under constant conditions (DD/WW, 17 °C)^28^ (Fig. 3A). The control and the *lin-42(ox461)* strains exhibited a *sur-5* promoter rhythm under cyclical and FR conditions, thereby providing additional evidence that nematodes can be synchronized through a dual cycle of light and temperature (Fig. 3C,D and Fig. S3B). Consistent with our previous experiments, the *lin-42(ox461)* mutant strain exhibited a longer period under constant conditions compared to the control strain (26.41 ± 0.47 h, n = 32 mutants vs. 24.4 ± 0.57 h, n = 37 control) (Fig. 3B). The *lin-42(ox461)* strain exhibited lower synchronization following this entrainment protocol compared to the control strain, as evidenced by the acrophase shift in the Rayleigh plots in LD/CW and DD/WW conditions, suggesting that a masking mechanism is involved (i.e., a direct effect of the zeitgeber on circadian rhythms) (Fig. 2F).

We then tested whether a transgene of the longest *lin-42b* isoform, which encodes both PAS and CK1BD domains, could rescue aberrant behavior in the *lin-42(ox461)* null mutant when overexpressed from an extrachromosomal array under its native promoter. Although this *lin-42b* transgene could not restore the period length to control values (Fig. 3B,E), it improved entrainment to the wild-type level (Fig. 3F). This partial rescue phenotype may be due to the nature of the extrachromosomal arrays, which induce high interindividual variability in gene expression. For *lin-42(n1089)* mutants that lack the PAS domain and *lin-42(ok2385)* mutants that lack the CK1BD domain, we did not find any significant differences in either period or synchronization compared to the control strain (Fig. S4). These results show that *lin-42* modulates the period of *sur-5-*driven luminescent rhythms and that either the PAS or the CK1BD domains may be sufficient for circadian entrainment in *C. elegans.*

### KIN-20 regulates the circadian period in adult nematodes

**Figure 4:**
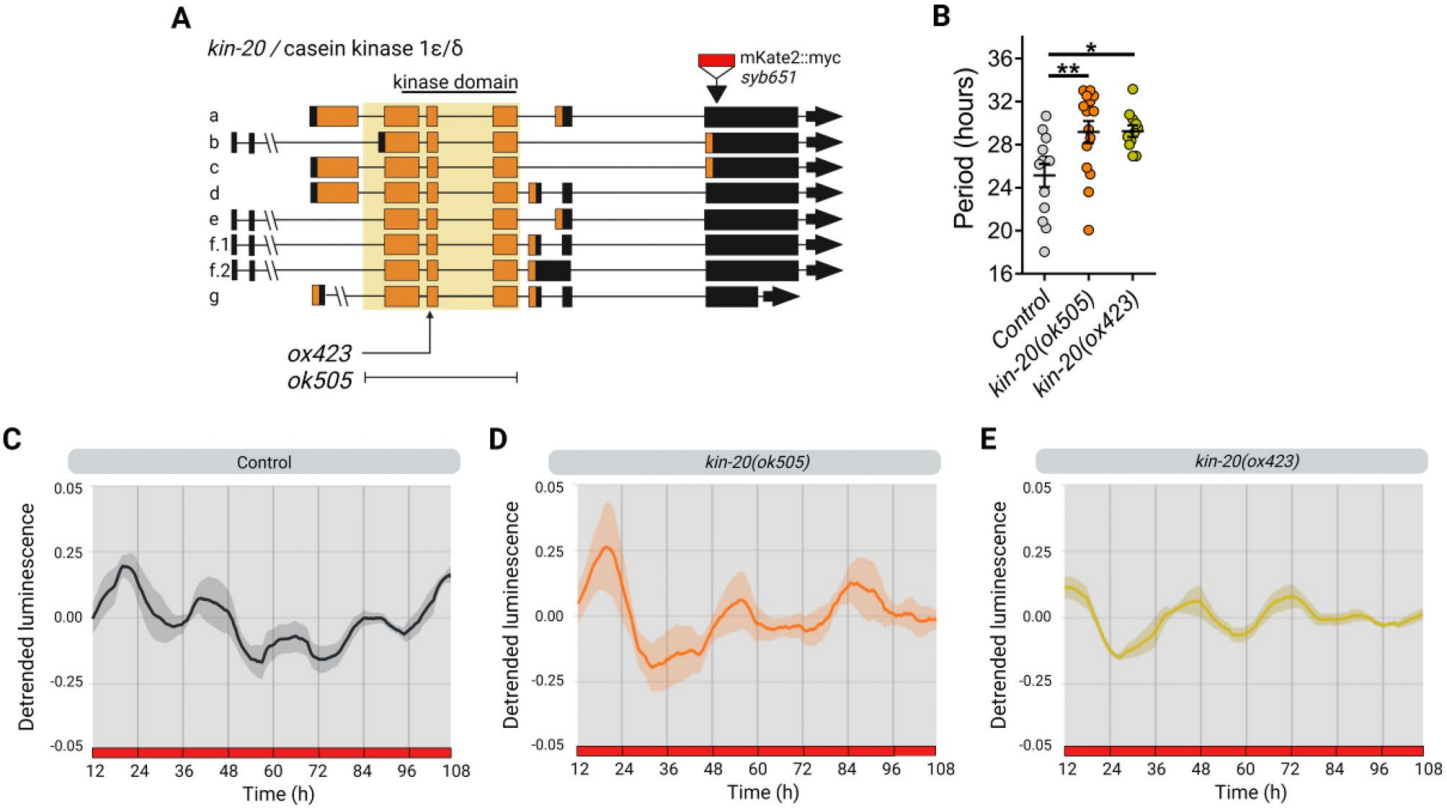
KIN-20 modulates period length in adult nematodes under FR conditions. (**A**) The *C. elegans kin-20* locus. The *ok505* allele harbors a deletion mutation in the kinase domain and the *ox423* allele is a nonsense mutation (Q344stop) in the kinase domain. The *syb651* allele is a knock-in of *mKate2::myc* in the 3ʹ UTR of isoform b. (**B**) Average endogenous period was determined for hours 18 h to 38 h for nematodes exhibiting a rhythm in FR. Error bars represent SEM. The average endogenous period of mutant strains of *kin-20* was compared with the control strain (25.15 ± 1.06 h, n = 13): *ok505* mutants (29.17 ± 1.01 h, n = 15) and *ox423* mutants (29.25 h ± 0.54 h, n = 11), *p = 0.011, **p = 0.0072, one-way ANOVA followed by Dunnett´s multiple comparisons test. The analysis includes three biological replicates for each strain. C-E. Average reporter activity of rhythmic adult populations under FR conditions (DD, 20 °C), control (**C**), *kin-20(ok505)* (**D**), and *kin-20(ox423)* (**E**). Luminescence signals are shown as mean ± SEM. The average reported activity from populations showing a similar first peak is shown. Representative single traces are shown in Fig. S5. Each population consisted of 50 adult nematodes per well.

To study the function of *kin-20* in the circadian system of *C. elegans*, we followed a similar approach as for *lin-42,* recording luminescence rhythms from two loss-of-function *kin-20* mutants (Fig. 4A), both of which are dumpy, have egg-laying defects, and show progressive paralysis^24^. We examined luminescence rhythms under constant conditions (DD/WW, dark and 20 °C) or under cyclic conditions (LD/CW, ~ 150/0 µmol/m^2^/s; 15.5 °C/17 °C) followed by constant dark and temperature conditions (DD/WW, dark and 17 °C). We first tested the null allele *ok505*, which has a complete deletion of the kinase domain (2201 bp deletion, lacking exons 3–5) and an out-of-frame insertion (Fig. 4A). Under constant conditions, *kin-20(ok505)* mutant animals showed a significantly longer period compared to the control strain (Fig. 4B–D and S5A-B, 29.17 ± 1.01 h, n = 15 mutant rhythmic, n total = 32, 47% rhythmic vs. 25.13 ± 1.06 h, n = 13 control rhythmic, n total = 24, 54% rhythmic). The *kin-20(ox423)* null mutant has a nonsense mutation (Q344stop) in the kinase domain that affects all isoforms (Fig. 4A). Similar to *ok505, ox423* mutants also showed a significantly longer period in luminescent rhythms compared to control worms (Fig. 4B–E and Fig. S5C, 29.25 h ± 0.54 h, n = 11 mutant rhythmic, n total = 24, 46% rhythmic vs. control).

**Figure 5:**
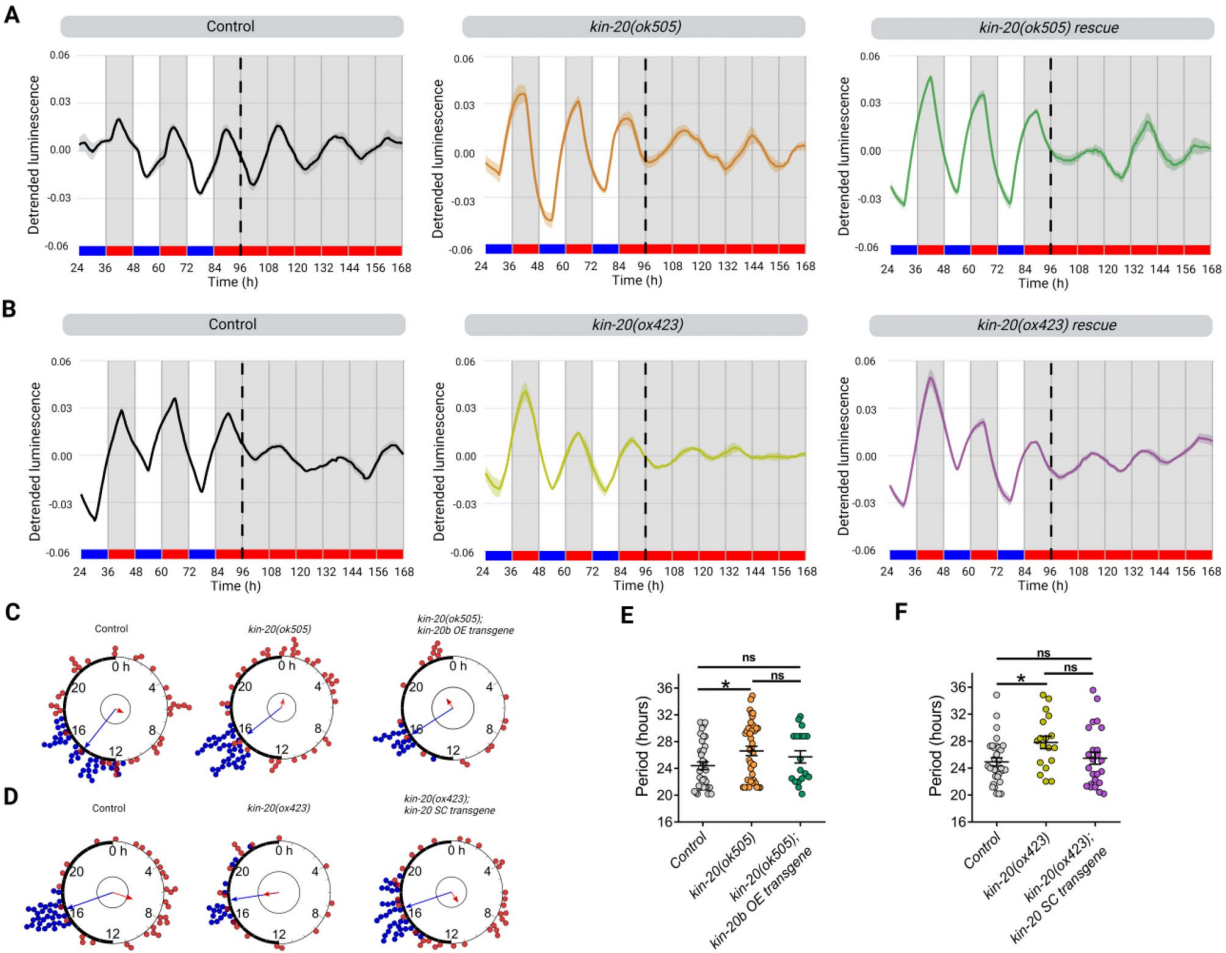
KIN-20 modulates period length in adult nematodes under cyclic conditions. (**A**, **B**) Representative luciferase activity rhythms of adult populations under dual cyclic conditions (LD/CW, ~ 150/0 µmol/m^2^/s; 15.5 °C/17 °C) and FR conditions (DD, 17 °C): control, *kin-20(ok505)*, *kin-20b* overexpression (OE) transgene (**A**) and control, *kin-20(ox423)* and *kin-20* single-copy transgene for all isoforms (**B**). Luminescence signals are shown as mean ± SEM. The average reported activity was displayed with rhythmic populations showing a similar first peak in FR. Each population consisted of 50 adult nematodes per well. The reported activity from all the wells for each strain is displayed in Fig. S6. (**C**, **D**) Rayleigh plots showing the phase of the bioluminescent peak under dual cyclic conditions (LD/CW, blue dots) and the first bioluminescent peak on the first day of release to FR (DD/WW, red dots) for rhythmic population nematodes: control (LD/CW: 14.55 ± 0.27 h, n = 37; R = 0.90 and DD/WW: 7.85 ± 0.85 h, n = 37; R = 0.07), *kin-20(ok505)* (LD/CW: 15.49 ± 0.12 h, n = 39; R = 0.97 and DD/WW: 22.83 ± 0.76 h, n = 39; R = 0.21), *kin-20b* overexpression (OE) transgene (LD/CW: 15.83 ± 0.18 h, n = 18; R = 0.97 and DD/WW: 2.03 ± 1.2 h, n = 18; R = 0.098) (**C**) and control (LD/CW: 16.7 ± 0.10 h, n = 32; R = 0.98 and DD/WW: 7.1 ± 0.76 h, n = 32; R = 0.35), *kin-20(ox423)* (LD/CW: 17.46 ± 0.37 h, n = 18; R = 0.91 and DD/WW: 17.43 ± 1.07 h, n = 18; R = 0.28) and *kin-20a/b/c* single-copy transgene (LD/CW: 16.08 ± 0.19, n = 24; R = 0.96 and DD/WW: 12.63 ± 0.94 h, n = 24; R = 0.25) (**D**). Arrows represent each group’s average peak phase of *sur-5::luc* expression (mean vectors for the circular distributions). The length of the vector represents the strength of the phase clustering, while the vector’s angle represents the mean phase. Individual data points are plotted outside the circle. The central circle represents the threshold for p = 0.05. (**E**) Average endogenous period of control (24.4 ± 0.57 h, n = 37), *ok505* mutants (26.61 ± 0.69 h, n = 39) and *kin-20b* overexpression (OE) transgene strain (25.73 ± 0.91 h, n = 18). One-way ANOVA followed by Tukey´s multiple comparisons test, *p = 0.0421; ns, p > 0.05. (**F**) Average endogenous period of control (24.93 ± 0.59 h, n = 32), *ox423* mutants (27.81 ± 0.92 h, n = 18) and *kin-20a/b/c* single-copy (CS) transgene (25.46 ± 0.89 h, n = 24). One-way ANOVA followed by Tukey´s multiple comparisons test, *p = 0.0365; ns, p > 0.05.

We further analyzed the effect of *kin-20* mutants on luminescence rhythms of the nematode under cyclical conditions, followed by release into free-running conditions. The *kin-20(ok505)* strain exhibited rhythmic *sur-5* promoter activity under both cyclical and in FR conditions, similar to the control strain. Again, *kin-20(ok505)* animals showed a longer period than the control strain in FR (Fig. 5A,E and Fig. S6A, 26.61 ± 0.69 h, n = 39 mutant vs. 24.4 ± 0.57 h, n = 37 control). Next, we asked if overexpression of the isoform b of KIN-20 (Fig. 4A), which has the highest homology to the kinase domain of CK1 ε/δ from mammals^9^, was able to rescue the longer period phenotype of *ok505* mutants. For this, we generated a strain with an extrachromosomal array overexpressing *kin-20b* under its native promoter. This resulted in a partial rescue of the period compared to wild-type animals (Fig. 5A,E and Fig. S6A, 25.73 ± 0.91 h, n = 18 rescue vs. 24.4 ± 0.57 h, n = 37 control), likely due to variable transgenic expression from extrachromosomal arrays, which induce high interindividual variability. All strains exhibited a similar dispersion of acrophases, showing low acrophase dispersion under cyclical conditions and higher acrophase dispersion in FR (Fig. 5C). Consistent with the phenotypes for *kin-20(ok505)*, *kin-20(ox423)* mutants also showed a significantly longer period compared to the control (Fig. 5B,F and Fig. S6B, 27.81 ± 0.92 h, n = 18 mutant vs. 24.93 ± 0.59 h, n = 32 control). We then attempted to rescue *kin-20(ox423)* behavioral defects by generating animals carrying a single copy of the transgene *oxSi1087* expressing RFP-tagged KIN-20, which includes all exons and 8.1 kb upstream of the first ATG codon (strain EG9581)^24^. These animals exhibited luminescence rhythms with a period length similar to wild-type controls (Fig. 5B,F and Fig. S6B, 25.46 ± 0.89 h, n = 24 rescue vs. 24.93 ± 0.59 h, n = 32 control). In addition, the ability to synchronize with the zeitgeber was comparable for all strains, as they displayed a similar dispersion of acrophases in LD/CW and DD/WW conditions (Fig. 5D). Therefore, our data demonstrate that *kin-20* is necessary for determining the period of circadian rhythms in *C. elegans* but is not involved in circadian entrainment.

### LIN-42b and KIN-20b regulate circadian rhythms in adult neurons

**Figure 6:**
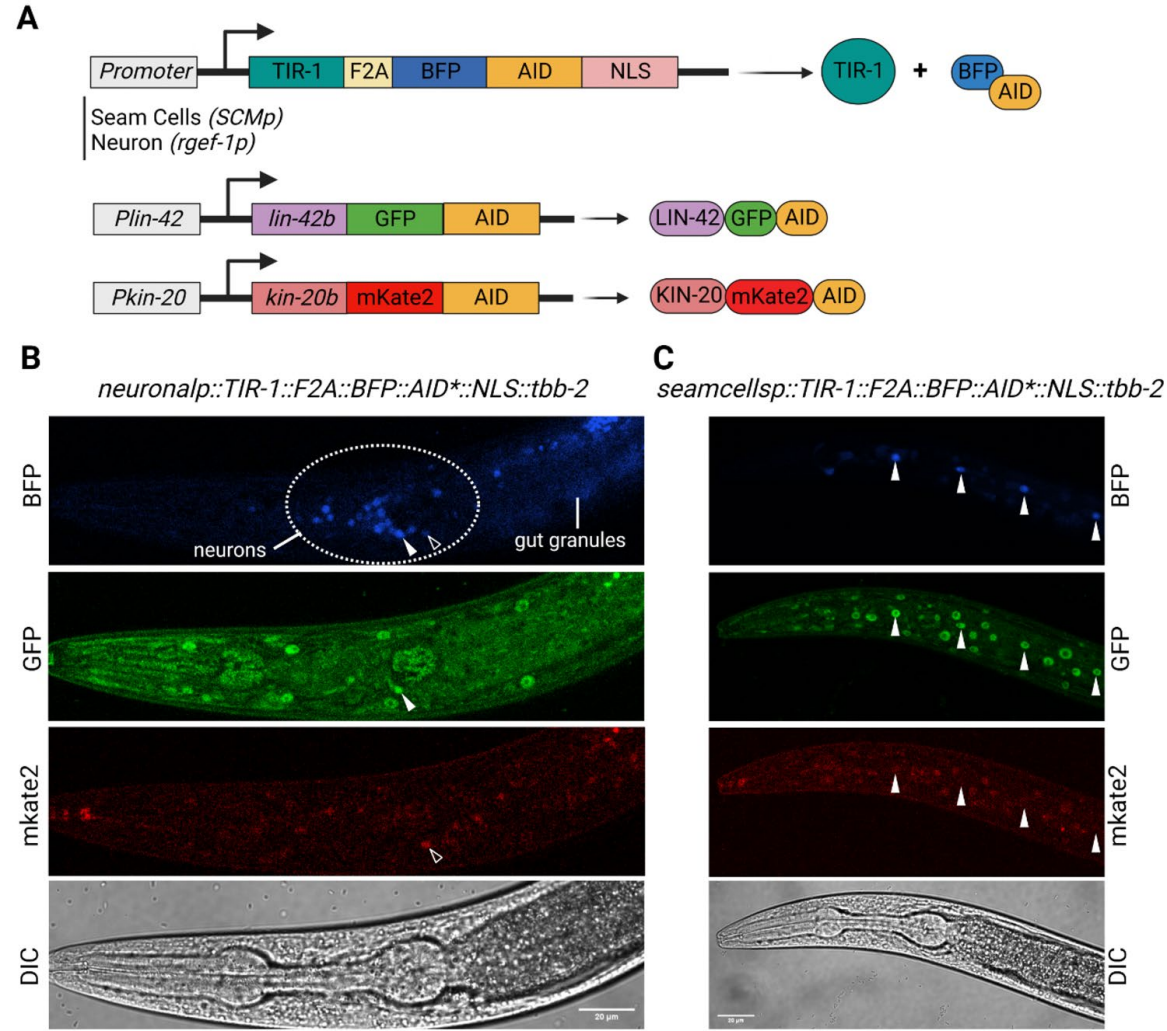
Expression of *lin-42b* and *kin-20b* in neurons and seam cells. (**A**) Schematic of the auxin-inducible degron (AID) system for *lin-42b* and *kin-20b*. TIR-1 is expressed under the promoter *SCMp* in seam cells and *rgef-1* in neurons. The TIR-1::F2A::BFP::AID*::NLS transgene cassette encodes for two separate protein products: TIR-1, which will interact with endogenous SCF proteins to produce an E3 ubiquitin ligase complex and can only bind the AID sequence in the presence of auxin; and an AID*-tagged BFP protein with a c-Myc nuclear localization signal (NLS) that functions as a readout for TIR-1 expression and internal control for TIR-1 activity. The sequence *lin-42b*::AID was tagged with GFP, and the sequence *kin-20b*::AID was tagged with mKate2. (**B**) GFP and mKate2 are detected in BFP-positive neurons (arrowheads) in L4 stage animals. C. GFP and mKate2 are detected in BFP-positive seam cells (arrowheads) in L4 stage nematodes. Signal in gut granules is autofluorescence. Scale bars represent 20 µm.

Although we have shown that both *lin-42* and *kin-20* are involved in circadian rhythm regulation in *C. elegans*, and since classical knock-out mutants show significant developmental defects, we cannot rule out the possibility of indirect developmental effects in the circadian phenotype of the mutants. To address this and gain more insight into the tissues from which both genes act to modulate period length, we used the auxin-inducible degradation (AID) system^40^. Using CRISPR, we generated *lin-42b/c*::GFP::AID*::3xFLAG and *kin-20b/c*::mKate2::AID knock-ins. We crossed both of these AID knock-ins into a strain expressing the *TIR1* transgene, either in seam cells or neurons, to drive the depletion of the targeted proteins, namely LIN-42 and KIN-20, in either cell type. These transgenes use a ribosomal 2A skip sequence to produce a nuclear-localized BFP::AID* reporter, which enables measurement of both transgene expression and TIR1 activity (Fig. 6A)^41^. We first analyzed the endogenous expression pattern of LIN-42B and KIN-20B in neurons that were labeled by *rgef-1p*::TIR-1::F2A::BFP::AID*::NLS::tbb-2 in L4 stage nematodes using confocal fluorescence microscopy. LIN-42B and KIN-20B can be observed in some neurons (Fig. 6B, Fig. S7A). Moreover, we generated a strain simultaneously carrying the two engineered knock-in loci, *lin-42b*::GFP::AID and *kin-20b*::mkate-2::AID, as well as the transgene *SCMp*::TIR-1::F2A::BFP::AID*::NLS::tbb-2, which expresses BFP in epidermal seam cells (Fig. 6A). We found that the protein LIN-42B is reliably detected in seam cells in L3/L4 stage larva (Fig. 6C, Fig. S7B) and that both LIN-42B and KIN-20B can be observed co-expressed in other epidermal cells. To further support this, we used the CeNGEN single-cell RNA sequencing dataset^42^, which provides gene expression profiles of all 302 neurons of the *C. elegans* nervous system in L4 nematodes^43^. We found that *lin-42* and *kin-20* are expressed in pharyngeal neurons, motor neurons, sensory neurons, and interneurons (Fig. S7C). Using scRNA-seq, recent studies have allowed the generation of a broad database to search for the expression of genes of interest in adult nematodes^44^. By using the WormSeq database, we also confirmed that, although at low levels, the genes *lin-42* and *kin-20* are both expressed in neurons and seam cells, among other cell types, in young adults (Fig. S7D). To gain more insights into *lin-42* and *kin-20* expression patterns, we performed qPCRs experiments to quantify the mRNA levels for the *lin-42b/c* and *kin-20b* genes from bulk RNA extractions from adult worms. Samples were taken every 4 h for one day under cyclic conditions (LD/CW) and for one day under constant conditions (DD/WW), starting from the young adult stage. Although no circadian pattern for either of the two genes was revealed, we did detect the expression of both genes throughout the experiment (S8A-B).

Subsequently, we asked whether LIN-42B and/or KIN-20B acts in the nervous system or seam cells to regulate circadian *sur-5p::luc* expression. First, we verified the degradation activity of TIR-1 in the tissues of interest under our luminescence recording conditions by following the fluorescence of BFP fused to TIR-1 (Fig. 6A, Fig. S9A,B). For neurons, L4-stage worms were used to detect BFP-positive cells (Fig. S9A). As seam cells are difficult to observe in adult nematodes, we used L3/L4 stage worms instead (Fig. S9B). As expected, we observed a significant loss of BFP fluorescence in neurons or seam cells after seven days of exposure to 4 mM auxin (K-NAA)^45^, confirming tissue-specific degradation by TIR-1 (Fig. S9). These results demonstrate that TIR-1 can efficiently deplete AID-tagged proteins in neuronal or seam cells under our luminescent recording conditions.

Next, we analyzed whether the fusion of GFP::AID or mKate2::AID alters the function of LIN-42B or KIN-20B, respectively. We measured transcription of the reporter gene *sur-5::luc::gfp* in animals with the *lin-42b*::GFP::AID and *kin-20b*::mKate2::AID constructs (with TIR-1 expressed in neurons or seam cells) and compared them with the control strain (*qvIs8*). We analyzed luminescence rates from L4 stage transgenic nematodes for three days under cyclic conditions (LD/CW, 15.5/17 °C, 12:12 h) and then for four more days under constant conditions (DD/WW, 17 °C). The transgenic nematodes expressing *lin-42b*::GFP::AID and *kin-20b*::mkate2::AID, crossed with either TIR-1 expressed in neuronal cells (Fig. S10A,B) or seam cells (Fig. S10C,D), showed similar behavior to the control strain in the absence of auxin. Thus, these results indicate that modifying the *lin-42* and *kin-20* alleles by the fusion of the fluorescent protein and the AID sequence and the expression of TIR-1 do not alter nematode rhythms.

**Figure 7:**
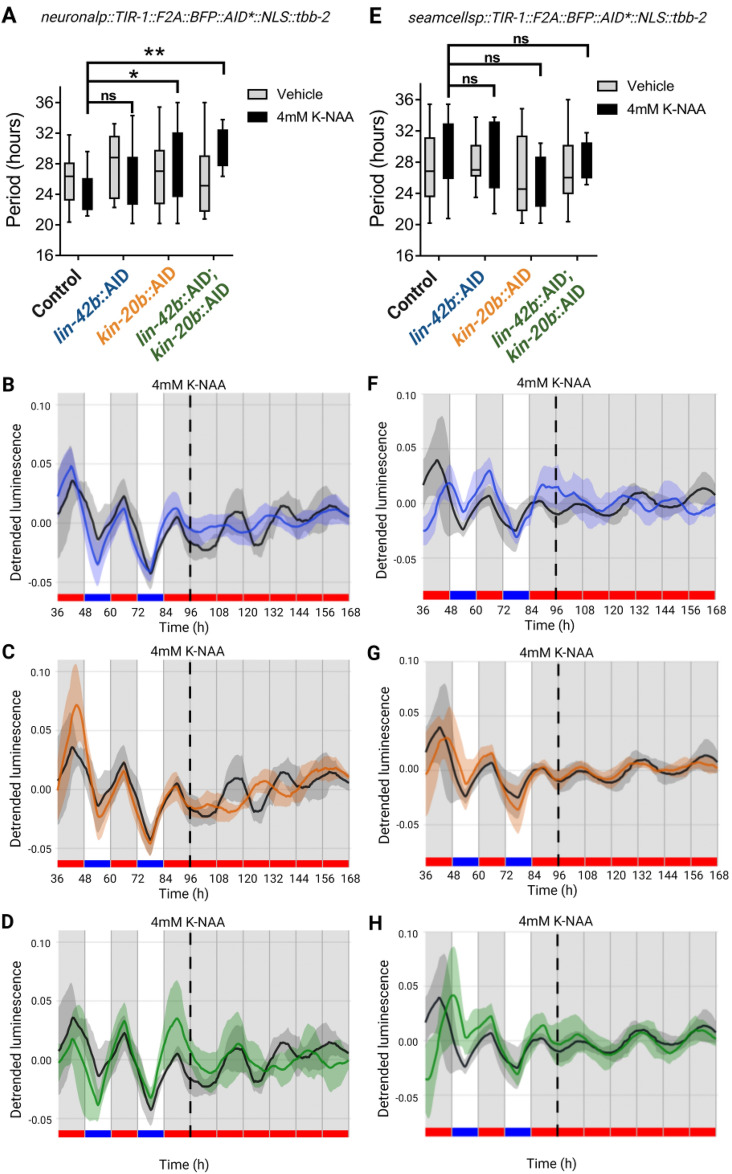
LIN-42b and KIN-20b regulate circadian rhythms in adult neurons. (**A**) Average endogenous period of adults nematodes that express *rgef-1p*::TIR-1::F2A::BFP::AID*::NLS::tbb-2 and the respective AID-tagged protein, exposed vehicle or 4 mM Auxin continuously throughout the luminescence experiment. Nematodes exposed to vehicle: Control (25.97 ± 0.85 h, n = 17), *lin-42b*::AID mutants (27.62 ± 1.14 h, n = 13), *kin-20b*::AID mutants (26.93 ± 1.30 h, n = 12) and *lin-42b*::AID; *kin-20b*::AID mutants (26.07 ± 0.92, n = 25). Nematodes exposed to 4 mM Auxin: Control (23.99 ± 0.86 h, n = 11), *lin-42b*::AID mutants (26.31 ± 0.89 h, n = 19), *kin-20b*::AID mutants (28.32 ± 1.12 h, n = 21) and *lin-42b*::AID; *kin-20b*::AID mutants (30.00 ± 0.70 h, n = 12). Two-way ANOVA followed by Dunnett's multiple comparisons test, *p = 0.0160, **p = 0.0020; ns, p > 0.05. (**B**–**D**) Representative luciferase activity rhythms of adult populations shown in E, exposed to 4 mM Auxin (K-NAA)*,* under the same dual cyclic conditions and FR conditions. Luminescence signals are shown as mean ± SEM. The average reported activity was displayed with rhythmic populations showing a similar first peak in FR. Each population consisted of 50 adult nematodes per well and the analysis includes three biological replicates for each strain. (**E**) Average endogenous period of adults nematodes that express *SMCp*::TIR-1::F2A::BFP::AID*::NLS::tbb-2 and the respective AID-tagged protein, exposed to vehicle or 4 mM Auxin continuously throughout the luminescence experiment. Nematodes exposed to vehicle: Control (26.96 ± 1.05 h, n = 18), *lin-42b*::AID mutants (27.87 ± 0.74 h, n = 14), *kin-20b*::AID mutants (26.16 ± 1.12 h, n = 17) and *lin-42b*::AID; *kin-20b*::AID mutants (26.88 ± 1.11, n = 16). Nematodes exposed to 4 mM Auxin: Control (28.87 ± 1.02 h, n = 18), *lin-42b*::AID mutants (28.86 ± 1.60 h, n = 8), *kin-20b*::AID mutants (25.44 ± 1.04 h, n = 10) and *lin-42b*::AID; *kin-20b*::AID mutants (27.93 ± 0.71, n = 11). Two-way ANOVA followed by Sidak's multiple comparisons test, ns, p > 0.05. (**F**–**H**) Representative luciferase activity rhythms of adult populations shown in (**E**), exposed to 4 mM Auxin (K-NAA)*,* under the same dual cyclic conditions and FR conditions. Luminescence signals are shown as mean ± SEM. The average reported activity was displayed with rhythmic populations showing a similar first peak in FR. Each population consisted of 50 adult nematodes per well and the analysis includes three biological replicates for each strain. The reported activity from all the wells for each strain is displayed in Fig. S11.

To examine the effect of depleting KIN-20 and LIN-42 in either seam cells or nervous system on the expression of *sur-5::luc::gfp*, we measured luminescence rates in adult transgenic nematodes for three days under cyclical conditions (LD/CW, 15.5/17 °C, 12:12 h) and then for four more days under constant conditions (DD/WW, 17 °C). We added 4 mM K-NAA in the luminescence medium on the first day of recording (after development) and compared the endogenous periods in transgenic nematodes exposed to drug or vehicle. Depletion of LIN-42 in neuronal cells, although showing a trend towards an altered period, did not generate a significant change in the period (Fig. 7A,B and Fig. S11A,B, 26.32 ± 0.89 h, n = 19 LIN-42::AID + 4 mM K-NAA vs. 24 h ± 0.86, n = 11 control + 4 mM K-NAA). This indicates that part of the effect found in *lin-42(ox461)* animals could be due to their developmental defects. However, we did observe a significant lengthening of the period in nematodes with neuronal depletion of KIN-20 (Fig. 7A,C and Fig. S11C,D, 28.32 ± 1.12 h, n = 21 KIN-20::AID + 4 mM K-NAA vs. 24 ± 0.86 h, n = 11 control + 4 mM K-NAA). These auxin-dependent circadian phenotypes in nematodes expressing TIR-1 in neuronal cells were similar to the phenotype of *kin-20(ok505)* and *kin-20(ox423)* mutants (Fig. 5). Depletion of both proteins (KIN-20 and LIN-42) in neurons also generated a significant change in period (Fig. 7A,D, 30.01 ± 0.70 h, n = 12 LIN-42/KIN-20::AID + 4 mM K-NAA vs. 24 ± 0.86 h, n = 11 control + 4 mM K-NAA). The most extended circadian period was observed when both KIN-20 and LIN-42 proteins were depleted, compared to nematodes where only KIN-20 or LIN-42 was depleted. Noteworthy, we did not observe any difference in the circadian period in worms depleted of LIN-42B and KIN-20B in seam cells, suggesting that the transcription of circadian *sur-5* is not regulated by KIN-20 or LIN-42 proteins in this cell type (Fig. 7E–H). Collectively, these data show that KIN-20 and LIN-42 regulate the period of *sur-5* luminescent rhythms in the nervous system of adult worms, independently of their developmental roles, consistent with post-transcriptional or post-translational regulation of a clock located in *C. elegans* neurons, as in other organisms^4,46^.

## Discussion

PER and CK1ε/δ proteins regulate circadian rhythms throughout metazoans^5,27^ and have homologous genes in *C. elegans*, LIN-42 and KIN-20, respectively. We examined whether these proteins also regulate *C. elegans’* circadian rhythms. We measured bioluminescence rhythms of the *sur-5* reporter gene, which allowed for continuous, non-invasive recording in the nematode^28^. Circadian regulation of the gene *sur-5* can be synchronized by a dual cycle of light and temperature and is maintained under constant conditions of light and temperature with a period close to 24 h while also exhibiting temperature compensation^28^, the three defining features of circadian rhythms.

A disruption in PER generates alterations in the period or eliminates rhythms in mice and flies^26,47–49^. Consistent with this, we found that the *lin-42(ox461)* null mutant lengthens the period of transcriptional rhythms at the *sur-5* promoter and produces a decrease in the percentage of nematodes that can be entrained by external stimuli, such as temperature and light. However, mutations exclusively in the PAS domains (*lin-42 (n1089)*) or the CK1BD domain (*lin-42 (ok2385)*) did not generate significant alterations in the endogenous period. Our observation that the *lin-42b* transgene did not rescue the long period of the null allele could be due to a mosaic expression of the transgene, which usually occurs in rescue assays of *C. elegans* mutants with extrachromosomal arrays^50^. In mice, *Per1* overexpression under a constitutive promoter lengthens the period of activity and body temperature rhythms^51^, suggesting that LIN-42 may have a distinct mechanism in timekeeping from its mammalian homologs.

The inclusion of different domains characterizes *lin-42* isoforms: *lin-42a* contains the CK1BD domain, *lin-42c* contains the PAS-A and PAS-B domains, and *lin-42b* has both^19^. During development, *lin-42b* and *lin-42a* play a more critical role than *lin-42c*^19,52^. We found that isoforms containing both PAS and CK1BD domains, like *lin-42b/d*, seem to have greater relevance in the circadian system of the adult nematode than isoforms containing only the PAS or the CK1BD domains, such as *lin-42c* or *lin-42a*, since specific deletions of these domains did not alter the endogenous period. This suggests that the different isoforms might regulate each other to generate the expression and proper function of *lin-42*; only deleting multiple domains induced an apparent alteration in circadian rhythms. This is different than in mammals, where an in-frame disruption of the PAS domain alone in *mPer2*^*Brdm1*^ mutant mice is enough to induce a significantly shorter period of locomotor activity and a loss of circadian rhythmicity in free running^38^. The *lin42b/c/a* isoforms exhibit transcriptional oscillations during developmental molt changes in *C. elegans* with ~ 8 h periods at 25 °C that are not temperature compensated^18^. In adult worms, previous studies did not find circadian transcriptional regulation of *lin*-*42* at the gene level under light/dark (LD) or cold/warm (CW) temperature cycles in whole-worm extracts^16,53,54^. Likewise, we did not detect a circadian transcription in *lin-42b* (nor in *kin-20*) mRNA during adulthood under our dual light and temperature entrainment condition. In this sense, it would be interesting to determine whether the expression of different *lin-42* isoforms, as happens for PER in other models, oscillates with a circadian pattern in adults in a specific group of cells. However, this would require long-read sc-RNAseq time-lapse experiments that are currently very challenging to do at the experimental level in *C. elegans*. Finally, it remains possible that *lin-42* expression is not circadian after development and that this gene functions in *C. elegans* in a novel way.

CK1ε/δ is important in maintaining the rhythmicity and period of the clock in mammals and other organisms^55,56^. Here, we show that the *kin-20(ok505)* and *kin-20(ox423)* mutants exhibit a significant period lengthening compared to the control strain without affecting entrainment to external cues. KIN-20 function can be partially rescued by overexpression of *kin-20b* in the null *kin-20(ok505)* mutant or by single-copy expression of all isoforms in *kin-20(ox423)*. This suggests that the function of KIN-20 is important for nematode development^8,23^ and neuronal stabilization^24^ and for maintaining rhythms in adult nematodes.

Similar to the rescue of the mutation in *lin-42(ox461)*, we believe that the rescue of the *kin-20(ok505)* mutation by overexpression of the gene *kin-20b* is partial because the transgenic expression is very variable among individual animals due to mosaicism of the extrachromosomal arrays. In addition, given the nature of the transgenes, it is likely that the expression from extrachromosomal arrays was also too high in comparison with the native levels needed to compensate for the effect. Whereas the fluorescence from the extrachromosomal arrays in the rescue lines was easily seen using our fluorescent dissecting microscope, we could not detect the fluorescent markers from our endogenously tagged *lin-42* and *kin-20* CRISPR strains without a confocal microscope. This indicates that the genes *lin-42* and *kin-20* are expressed at very low levels in vivo. Further, there is also the possibility of different isoforms playing specific roles in period regulation. In the case of *kin-20(ox423)*, we tried to solve the dosage and inter-individual variability expression issue with the single copy rescue coding for all isoforms. Although this partially rescued the period length, more was needed to show statistical significance. This means that maybe more than one isoform is involved in period regulation. Future experiments, including rescues with overexpression of the other isoforms, are needed to elucidate their function fully.

In mammals, CK1ε/δ stably binds to PER and phosphorylates it to regulate its stability via the proteasome^6,39,57^. While this particular interaction has not yet been demonstrated in *C. elegans*, it was recently shown that KIN-20 regulates the expression of LIN-42A in larval stages L3 and L4^23^. Here, we show that LIN-42b and KIN-20b are expressed in neuronal cells and in seam cells. However, more research is needed to determine the possible role of other nematode casein kinases in regulating LIN-42 and a possible interaction between the proteins.

To rule out the contribution of the significant developmental defects shown by *lin-42* and *kin-20* loss of function mutants on the circadian phenotypes found and to also study the site of action of KIN-20 and LIN-42 needed to sustain circadian rhythms, we depleted these proteins in a tissue-specific manner using the AID system. Remarkably, we found a significantly more extended period in the *sur-5* luminescent rhythm when KIN-20 or both LIN-42 and KIN-20 were depleted in neuronal cells but not in epidermal seam cells. Since LIN-42 neuronal depletion alone was not enough to fully recapitulate the more extended period found in *lin-42(ox461)* null mutants, this could indicate that part of the mutant effect could be attributed to developmental defects shown by these mutant animals. The exacerbated period change when depleting both proteins may be because the interaction between LIN-42 and KIN-20 is functionally different from mammals or that KIN-20 could regulate other proteins in nematodes that also participate in circadian rhythms.

In summary, we show that the PERIOD and CK1ε/δ clock homologs LIN-42 and KIN-20 play a vital role in the generation of circadian rhythms in adult nematodes, mainly by regulating period length. Their evolutionary and structural conservation of key domains and the dimerization strategy for LIN-42 suggest that there may be some shared aspects of nematode and mammalian clocks. In addition, our results highlight and support the existence of an ancestral circadian clock in metazoans that relates developmental regulation and adult behavior in the nematode, strengthening its use as an exciting model system in chronobiology to explore the molecular origins of metazoan timekeeping.

## Materials and methods

### Strains and maintenance

*C. elegans* strains were cultured as previously described^26^. All strains were maintained under a dual cycle of light and temperature, Light:Dark (LD, ~ 150/0 µmol/m^2^/s) and Cold:Warm (CW, 18.5/20 °C) cycles 12:12 h, LD and CW were established in the best phase combination, as already described^28^. *C. elegans* hermaphrodites were analyzed.

Zeitgeber (i.e., “time giver” or entraining agent) time 0 or ZT0 (9:00 a.m.) indicates the time of lights on and the cold phase. Circadian Time (CT) refers to a specific time in the free running conditions (constant darkness, DD, and warm constant temperature, WW). Photo and thermal conditions were controlled with an I-291PF incubator (INGELAB, Argentina) and temperature was monitored using DS1921H-F5 iButton Thermochrons (Maxim Integrated, USA). Four cool white LED light strips (approx.150 µmol/m^2^/s) were used as the light source for the luminescence recordings.

The mutant and transgenic strains used in this study are listed in Supplementary Table 2. Strains VC398 *kin-20(ok505)*X outcrossed × 1, MT2257 *lin-42(n1089)*II outcrossed × 0, and RB1843 *lin-42(ok2385)*II outcrossed × 0 were provided by the Caenorhabditis Genetics Center (CGC), University of Minnesota, USA. Strains RG1590 *lin-42(ox461)*II outcrossed × 3 and plasmids pCP2 [*plin-42b/c::isoformb::gfp:unc-54*], pMJ13 [*plin-42b/c::lin42c::gfp::unc54*] and pHG82 [*plin-42a::lin42a::gfp::unc54*] were donated by the Ann E. Rougvie Lab^17^. Strains EG5202 *oxIs12* [*punc-47::gfp, lin-15(*+*)*] *kin-20(ox423)*X, and EG9581 *oxSi1087* [*pkin-20::rfp::kin-20::kin-20 3'UTR, Cb unc-119(*+*) ∗ ttTi5605*] II; *oxIs12* [*punc-47::gfp, lin-15(*+*)*] *kin-20(ox423)*X were provided by Erik M. Jorgensen Lab^24^, and were later crossed with the VQ1310 *qvIs8* strain, generating the DG1 and DG2 strains, respectively. Strain VQ1850 *kin-20*(*syb4198*[*kin-20b/c::mkate-2^degron::HA*])X was constructed by Sunybiotech (Fuzhou, China). The strains JDW136 *lin-42*(*wrd35*[*lin-42::GFP^degron::3xFLAG*])II, JDW233 [wrdSi46SCMp*::TIR1::F2A::BFP::AID*::NLS::tbb-2 3'UTR]I, DV3805 [eSi7(*rgef1p::TIR1::F2A::mTagBFP2::NLS::AID::tbb-2 3’UTR*]I were generated as described^58^.

Transgenic animals were generated by standard microinjection techniques^59^ and the integration of *psur5::luc::gfp* was induced by UV radiation to generate *qvIs8*^60^. Subsequently, seven crosses were performed to clean the strain of unwanted mutations.

### Molecular constructs

To obtain the promoter of the gene *kin-20*, 2.6 kb upstream of the start codon of the isoform b of the gene *kin-20* was amplified by PCR from fosmid WRM0617dH07 (Supplementary Table 3). A 1045 bp DNA fragment corresponding to the isoform b of *kin-20* was obtained by PCR from genomic DNA of wild-type nematodes (Supplementary Table 3). Subsequently, the fragment was digested with the restriction enzymes XbaI and KpnI (NEB) and cloned into vector *punc-122::dsRed* (*coel::RFP* Plasmid #8938, Addgene) to generate *punc-122::isoform-b::dsRed* (6.8 kb). The *kin-20* promoter (2647 bp) was cloned into the previously generated plasmid (*punc-122::isoform-b::rfp*) by digestion with restriction enzymes SphI and XbaI to generate the *pkin-20::isoform b::rfp* (7887 bp) construct.

### Luminescence assays

For all assays, nematodes were maintained in LD/CW 12:12 h. Adult nematodes were treated with the alkaline hypochlorite solution method to obtain synchronized populations of embryos^61^. The harvested embryos were cultured overnight in a 50 mL Erlenmeyer flask with 3.5 mL of M9 buffer (42 mM Na_2_HPO_4_, 22 mM KH_2_PO_4_, 85.5 mM NaCl, 1 mM MgSO_4_), 1X antibiotic–antimycotic (Thermo Fisher Scientific) and 10 μg/mL of tobramycin (Tobrabiotic, Denver Farma) at 110 rpm in LD/CW (~ 150/0 µmol/m^2^·s); 18.5/20 °C, Δ = 1.5 °C ± 0.5 °C) conditions. The next day, L1 larvae were placed on a Petri dish with NGM (0.3% NaCl, 0.25% peptone, 5 μg/mL cholesterol, 1 mM CaCl_2_, 1 mM MgSO_4_, and 1.7% agar in 25 mM potassium phosphate buffer, pH 6.0) and bacterial lawns of *Escherichia coli* strain HB101.

Two days later, L4 stage nematodes were picked onto 96-well plates (50 nematodes/well). They were visualized on a SMZ100 stereomicroscope equipped with an epi-fluorescence attachment (Nikon) with a cool Multi-TK-LED light source (Tolket) to avoid plate warming. They were washed once with M9 buffer to remove all traces of bacteria and resuspended in 200 μL of luminescence medium. Luminescence medium contained Leibovitz's L-15 media without phenol red (Thermo Fisher Scientific) supplemented with 1X antibiotic–antimycotic (Thermo Fisher Scientific), 40 μM of 5-fluoro-2′-deoxyuridine (FUdR) to avoid new eclosions, 5 mg/mL of cholesterol, 10 μg/mL of tobramycin (Tobrabiotic, Denver Farma), 1 mM of d-luciferin (Gold Biotechnology) and 0.05% Triton X-100 to increase cuticle permeabilization. Unless otherwise specified, all chemical compounds were bought from Sigma-Aldrich (St. Louis, MO).

For the luminescence assays in LD/CW and FR, luminescence from nematode populations (50 nematodes) was measured using a Berthold Centro LB 960 microplate luminometer (Berthold Technologies) stationed inside an incubator (INGELAB) to allow tight control of the light and temperature in each experiment. Microwin 2000 software version 4.43 (Mikrotek-Laborsysteme) was programmed to leave the plate outside the luminometer after each recording to expose nematodes to environmental cues. The luminescence of each well was integrated for 10 s every 30 min. The scheme used for all experiments was three days at a 12/12 h LD/CW cycle (~ 150 µmol/m^2^/s/0; 15.5/17 °C; ZT0, lights on and onset of the cold-temperature phase) and four days at the FR condition (DD/WW, dark/17 °C). This allows us to do long recordings (eight to ten days) and analyze synchronization and entrainment. In the assays only in FR conditions, we measured the luminescence from nematode populations (50 nematodes per well) with an AB-2550 Kronos Dio luminometer. At ZT12, the plates were transferred to the Kronos luminometer and monitored in DD/WW (dark/20 °C, minimal temperature permitted in this luminometer) for seven days. Recordings from nematode populations were taken every 30 min with an integration time of 1 min.

### Data acquisition and analysis

Luminescence was sampled at 30-min intervals. Background noise was extracted from the raw data obtained from the luminometer. In all cases, the first 12–24 h of recording were removed due to accumulation of the luciferase enzyme. All raw data were analyzed using a Shiny app developed in the laboratory (https://ispiousas.shinyapps.io/circaluc/). The raw data were detrended, smoothed, and normalized to the initial maximum value of each sample and plotted using R (R Core Team, 2021). All data are shown as mean ± SD or SEM of luminescence as indicated in the figures. In each case, the mean corresponds to a population of nematodes (50 nematodes). Subsequently, the circadian period was calculated from the population data using the Lomb-Scargle (LS) periodogram using the lomb R package^62^, within a range of 18–35 h and with an oversampling of 30. The acrophase (time at peak) and amplitude of each signal was estimated using the Cosinor method by fitting a cosine waveform to the data using a non-linear least squares regression implemented through the NLS function of base R and obtaining the R^2^ of the fit. Any signal resulting from population analysis with a 24-h period and an R^2^ adjustment ≥ 0.5 was considered “Synchronized” under cyclic training conditions. In the case of free-running rhythms, any signal resulting from the analysis with a period range between 18 and 35 h, and an R^2^ adjustment ≥ 0.5 was considered “Circadian”. For statistical analyses, the GraphPad Prism 7 software was used. Statistical significance was set at alpha = 0.05. Final figures were generated using Biorender (https://app.biorender.com/).

### Fluorescence microscopy assays

Nematodes were maintained under cyclic light and dark and temperature conditions (LD/CW, 12:12 h) as described in the strains and maintenance section. For the co-expression analysis of LIN-42 and KIN-20 proteins, nematodes in the L4 stage/young adults were taken from the NGM plate with bacteria and washed twice with M9 buffer. The animals were mounted on a 5% agarose pad and paralyzed with 2% azide. Fluorescent images of Fig. 6B,C were captured using a Nikon A1R confocal microscope with a 60 × oil immersion objective. All other images were taken using a Leica laser-scanning spectral confocal microscope TCS SP8 (Leica), with a scale of 40X. Lasers to excite GFP, mKate2, and BFP, respectively, were used and two independent experiments were performed with at least ten nematodes. Image preparation and co-expression analyses were done using ImageJ.

### Protein depletion using the auxin-inducible degron (AID) system

Nematodes were maintained under the same conditions explained in the luminescence assay section. We added 4 mM of the auxin K-NAA (Phyto-Technology Laboratories, N610)^63^ into the luminescent medium on the first day of the assays (ZT = 12, Time = 0). Controls for experiments were performed using L15 (Leibovitz's L-15 Medium, vehicle) with an equivalent volume. BFP positive cells were measured in nematodes exposed to the drug or the vehicle control for seven days to check the prolonged effect of auxin. L4 stage nematodes were used to observe neurons, and L2/L3 stage nematodes were used to observe seam cells. Animals were mounted on a 5% agarose pad and immobilized with 2% azide. All images were taken with a Leica TCS SP8 laser scanning spectral confocal microscope with a 40X scale. Two independent experiments were performed with at least ten nematodes each.

### RNA extraction and real-time PCR

Populations of L1 nematodes of the strain N2 (Bristol) were synchronized by the chlorine technique and grown on NGM plates with bacteria (*E. coli* HB101) under a dual cycle of light and temperature 12 h: 12 h (~ 150 µmol/m^2^ s:18.5 °C/0 µmol/m^2^ s:20 °C), up to the stage L4. Subsequently, they were collected at ZT4, washed with M9 buffer to remove the remains of bacteria, and passed through a 4 Erlenmeyer, with luminescence medium without luciferin. Nematodes were entrained under a dual light and temperature cycle 12 h:12 h (~ 150 µmol/m^2^/s:15.5 °C/0 µmol/m^2^/s:17 °C), shaken at 110 rpm for two days and then for two more days in constant conditions (darkness, 17 °C). Four independent biological samples (n = 4) of ~ 4000 nematodes each were collected every four h, starting at ZT1 on the second day under cyclic conditions and under constant conditions. According to the manufacturer's instructions, total RNA was isolated using 200 µL of TRIzol reagent (Life Technologies). RNA solutions were quantified using NanoDrop1000 (Thermo Fisher Scientific), and their integrity was evaluated by electrophoresis. Two µg of total RNA were treated with DNAse I amplification grade (Thermo Fisher Scientific), and complementary DNA (cDNA) was synthesized using oligo(dT) primers and the MLLV reverse transcriptase (PB-L). Gene amplification was performed on a QuantStudio 3 PCR instrument (Thermo Fisher Scientific), using 10 µL of final reaction volume containing 1 µL of cDNA as the template, 1 × of the SYBR Green PCR Master Mix 3.0 (PB-L), and the corresponding primers at a final concentration of 0.4–0.6 µM. The cDNA template was amplified in duplicate, with the following conditions: 95 °C for 10 min, followed by 45 cycles of 95 °C for 15 s and 60 °C for 1 min. Relative gene expression was calculated using the 2^−∆∆Ct^ method, and Y45F10D.4 was the reference housekeeping gene.

The primers used for amplification were, Fw-lin42b/c: 5ʹ-CCGAAAAATGGAGCTAGTCG-3ʹ, Rv-lin42b/c: 5ʹ-CGAAAGTCTTCGCCATAACC-3ʹ, Fw-kin-20b: 5ʹ-CTGGAACTGCAAGATACGCC-3ʹ, Rv-kin-20b: 5ʹ-CGGGAGAGTTCCACGATTAAAG-3ʹ, Fw-Y45f10D.4: 5ʹ-GTCGCTTCAAATCAGTTCAGC-3ʹ, Rv-Y45f10D.4: 5ʹ-GTTCTTGTCAAGTGATCCGACA-3ʹ.

### Expression and purification of recombinant protein

The N-terminus of LIN-42 (residues 1–315 or 41–315) was expressed from a pET22-based vector in *Escherichia coli* Rosetta2 (DE3) cells based on the Parallel vector series^64^. The protein was expressed downstream of an N-terminal TEV-cleavable HisGβ1 tag that leaves the sequence ‘GAMDPEF’ on the N-terminus of LIN-42 after TEV cleavage. Cells were grown in LB media at 37 °C until the O.D._600_ reached ~ 0.8, and then protein expression was induced with 0.5 mM IPTG, and cultures were grown for an additional ~ 18 h at 18 °C. Cells were centrifuged and resuspended in 50 mM Tris, pH 7.5, 300 mM NaCl, 20 mM imidazole, 5% glycerol, 1 mM tris(2-carboxyethyl)phosphine (TCEP), and 0.05% Tween-20. For purification, cells were lysed with a microfluidizer followed by sonication, and the lysate was clarified via centrifugation. Ni–NTA affinity chromatography was used to extract HisGβ1-LIN-42 from the lysate and the HisGβ1 was cleaved using His-TEV protease overnight at 4 °C in a low imidazole buffer. The cleaved protein was then separated from the tag and TEV by a second Ni–NTA affinity column and further purified using size exclusion chromatography on a HiLoad 16/600 Superdex 75 prep grade column (GE Healthcare) in 50 mM Tris, pH 7.5, 200 mM NaCl, 1 mM EDTA, 5% glycerol, 1 mM TCEP, and 0.05% Tween-20. Small protein aliquots were frozen in liquid nitrogen and stored at − 70 °C.

### Limited proteolysis and mass spectrometry of recombinant protein

Limited proteolysis of purified protein was performed at 1.5 mg/mL in 25 mM HEPES pH 7.5, 200 mM NaCl and 5 mM DTT with sequencing-grade trypsin (Promega) for one hour at room temperature with 1:800 or 1:1600 mass (w/w) ratios with trypsin for the indicated timepoints. Reactions were quenched with the addition of an equal volume of 2X SDS Laemmli buffer (Bio-Rad) and samples were boiled at 95 °C for 5 min. Digested fragments were resolved by 20% SDS-PAGE and visualized by Coomassie stain. Samples for mass spectrometry were quenched by the addition of formic acid to a final concentration of 1% (v/v). Samples were desalted and separated by HPLC (Surveyor, Thermo Finnegan) on a Proto 300 C4 reverse-phase column with 100 mm × 2.1 mm inner diameter and 5 μm particle size (Higgins Analytical, Inc) using a mobile phase consisting of Solvent A: 0.1% formic acid in HPLC grade water and Solvent B: 0.1% formic acid in acetonitrile. The samples were analyzed using an LTQ Orbitrap linear ion trap mass spectrometer system (Thermo Finnegan). Proteins were detected by full scan MS mode (over the m/z 300–2000) in positive mode with electrospray voltage set to 5 kV. Mass measurements of deconvoluted ESI mass spectra of the reversed-phase peaks were generated by Magtran software.

### Crystallization and structure determination

Crystallization was performed by hanging-drop vapor-diffusion method at 22 °C by mixing an equal volume of LIN-42 N-terminus residues 41–315 (4 mg/mL) with a reservoir solution of 150 mM lithium sulfate and 1.5 M sodium potassium tartrate. Hanging drops were also seeded using a cat whisker dipped in crushed LIN-42 crystals obtained from the conditions above, but in a protein:reservoir ratio of 2:1. The crystals were looped and briefly soaked in a drop of reservoir solution with 20% glycerol, as a cryopreservant, and then flash-cooled in liquid nitrogen for X-ray diffraction data collection. Data sets were collected at the APS beamline 23-ID-D. Data were indexed, integrated, and merged using the CCP4 software suite^65^. Structures were determined by molecular replacement with Phaser MR^66^ using the mouse PER2 PAS-B domain (PDB: 3GDI). Model building was performed with Coot^67^, and structure refinement was performed with PHENIX^68^. All structural models and alignments were generated using PyMOL Molecular Graphics System 2.5.1 (Schrödinger, LLC).

### Supplementary Information

Supplementary Information.

## Data Availability

Data is provided within the manuscript or supplementary information files. In addition, raw data for all results reported in the article are fully available upon request. Please contact Dr. Melisa Lamberti (melisalamberti@gmail.com) or Dr. Diego Golombek (dgolombek@gmail.com) for further information.
