# Supplementary material for: Clock gene homologs lin-42 and kin-20 regulate circadian rhythms in C. elegans
Source: Sci Rep. 2024 Jun 5;14:12936. doi: 10.1038/s41598-024-62303-9 (PMC11153552; doi:10.1038/s41598-024-62303-9)
Supplement: Supplementary file 1 — Supplementary Information. [file 41598_2024_62303_MOESM1_ESM.pdf]

Lamberti et al, Supplementary information

Figure S1. Structural characterization of the LIN-42 N-terminus. A. Limited trypsin proteolysis of LIN-42 residues 1-315 at the indicated mass ratios (trypsin:protein) for the indicated timepoints. **B.** Liquid chromatography/mass spectrometry analysis of limited trypsin proteolysis of LIN-42 with calculated masses of the corresponding fragments. **C.** Secondary structure prediction of LIN-42 residues 1-315 (JPred) with domain predictions. **D.** Structural alignment of the LIN-42 PAS-B (PDB 8GCI, gray) with the AlphaFold model (https://alphafold.ebi.ac.uk/entry/Q65ZG8) colored by the model confidence. **E.** Predicted aligned error of the LIN-42 AlphaFold model. The shade of green at position (x, y) indicates the expected position error at residue x when the predicted and true structures are aligned on residue y.

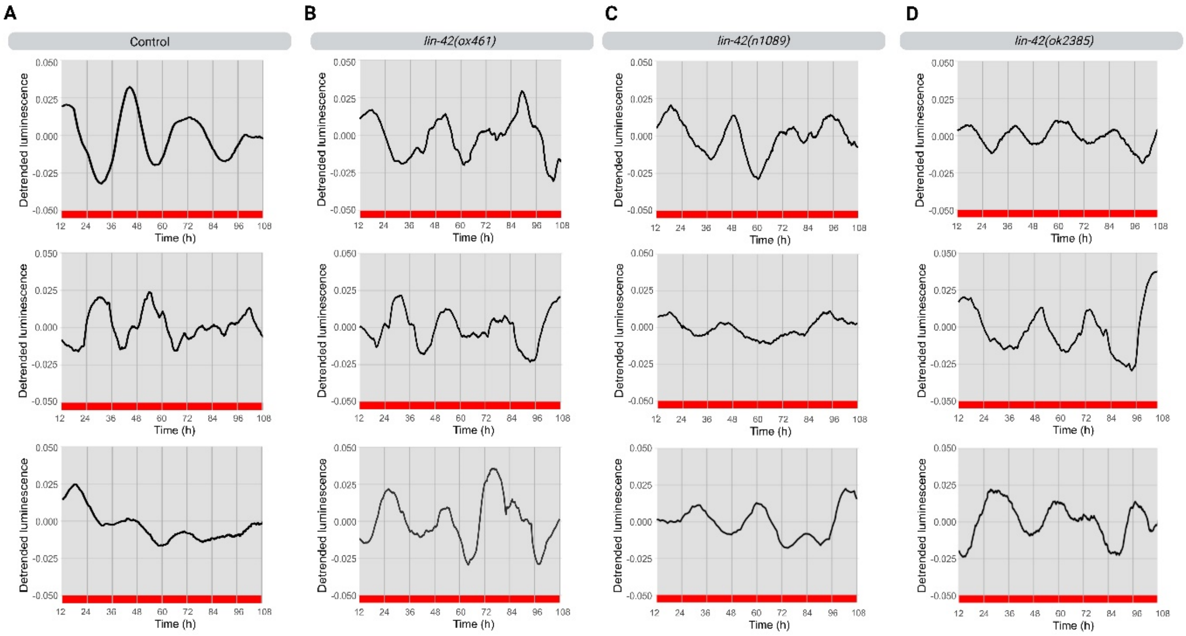

Figure S2. Circadian rhythms occur in *lin-42* mutants under FR. Representative single traces of luciferase activity rhythms of adult populations shown in control (**A**), *lin-42(ox461)* (**B**), *lin-42(n1089)* (**C**), *lin-42(ok2385)* (**D**), under FR conditions.

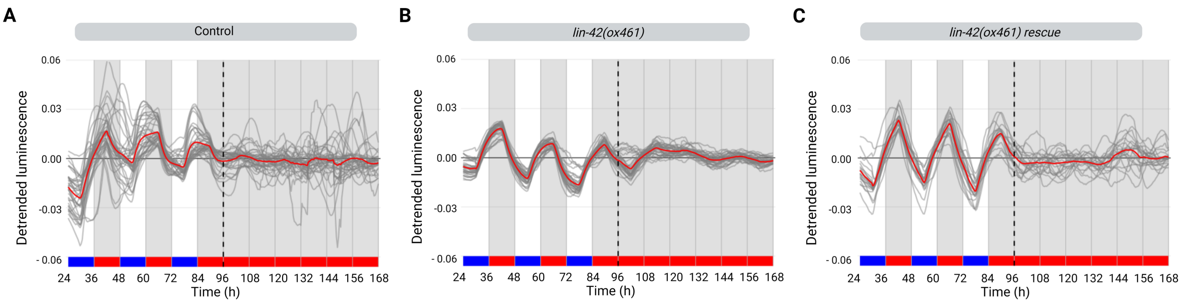

Figure S3. *lin-42* mutants are rhythmic under cyclic and FR conditions. Representative luciferase activity rhythms of adult populations under dual cyclic conditions (LD/CW, ~150/0 μmol/m^2/s; 15.5°C/17°C) and FR conditions (DD, 17°C): control (**A,** n=37), *lin-42(ox461)* (**B**, n=32), *lin-42b* overexpression (OE) transgene strain (**C**, n=20). Luminescence signals are shown as mean ± SEM in red line and all the individual wells are represented in the gray line. Each population consisted of 50 adult nematodes per well. The analysis includes three biological replicates for each strain.

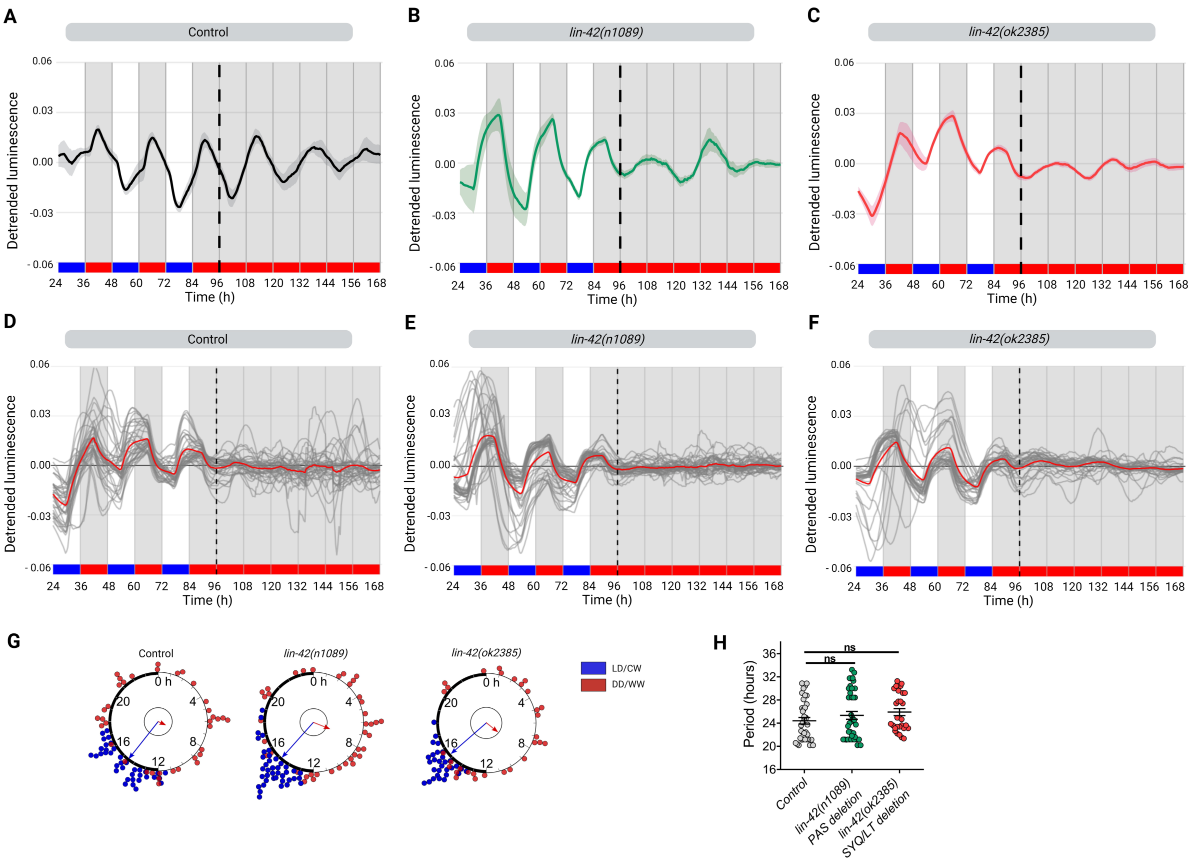

Figure S4. Circadian rhythms in *lin-42(n1089)* and *lin-42(ok2385)* strains. A-C. Representative luciferase activity rhythms of adult populations under dual cyclic conditions (LD/CW, ~150/0 μmol/m^2/s; 15.5°C/17°C) and FR conditions (DD, 17°C), control (**A**), *n1089* mutants (**B**) and *ok2385* mutants (**C**). Luminescence signals are shown as mean ± SEM. The average reported activity was displayed with populations

showing a similar first peak in FR conditions. Each population consisted of 50 adult nematodes per well. **D-F.** Average reporter activity of rhythmic adult populations under dual cyclic conditions and FR conditions, control (**D**, n=37), *n1089* mutants (**E**, n=40) and *ok2385* mutants (**F**, n=30). Luminescence signals are shown as mean ± SEM in red line and all the individual wells are represented in the gray line. The analysis includes three biological replicates for each strain. **G.** Representation of the acrophase distribution in Rayleigh plots under dual cyclic conditions (LD/CW, blue dots) and FR (DD/WW, red dots) for rhythmic population nematodes: control (LD/CW: 14.55 ± 0.27 h, n=37; R=0.90 and DD/WW: 7.85 ± 0.85 h, n=37; R=0.07), *lin-42(ok2385)* mutants (LD/CW: 15.24 ± 0.17 h, n=30; R=0.96 and DD/WW: 8.63 ± 0.87 h, n=30; R=0.20) and *lin-42(n1089)* mutants (LD/CW: 14.79 ± 0.20 h, n=40; R=0.94 and DD/WW: 7.52 ± 0.72 h, n=40; R=0.27). Arrows represent the average peak phase of *sur-5::luc* expression (mean vectors for the circular distributions) of each group. The length of the vector represents the strength of the phase clustering while the angle of the vector represents the mean phase. Individual data points are plotted outside the circle. The central circle represents the threshold for p=0.05. **H.** Average endogenous period of *lin-42* mutants vs control: control (24.4 ± 0.56 h, n=37), *n1089* mutants (25.34 ± 0.67 h, n=40), *ok2385* mutants (25.91 ± 0.60 h, n=30). One-way ANOVA followed by Dunnett´s multiple comparisons test, ns, p>0.05.

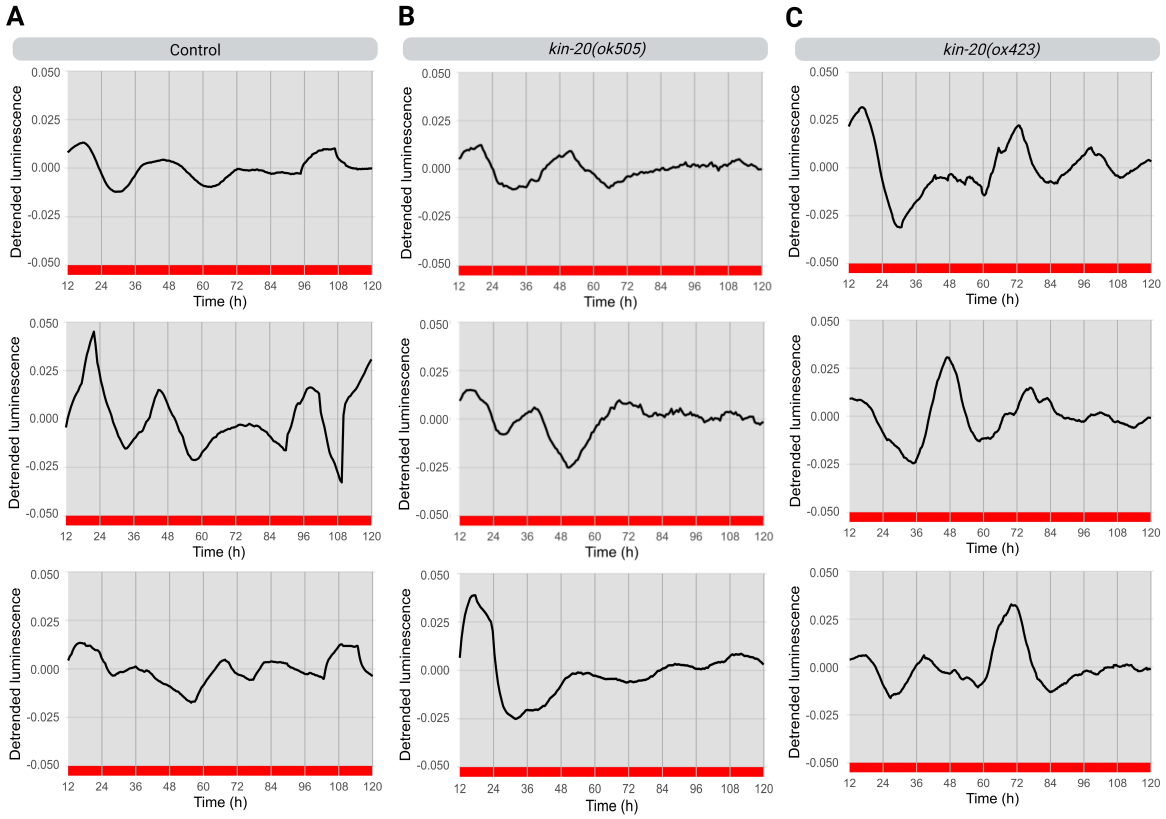

Figure S5. Circadian rhythms occur in *kin-20* mutants under FR. Representative single traces of luciferase activity rhythms of adult populations shown in control (**A**), *kin-20(ok505)* (**B**), *kin-20(0x423)* (**C**), under FR conditions.

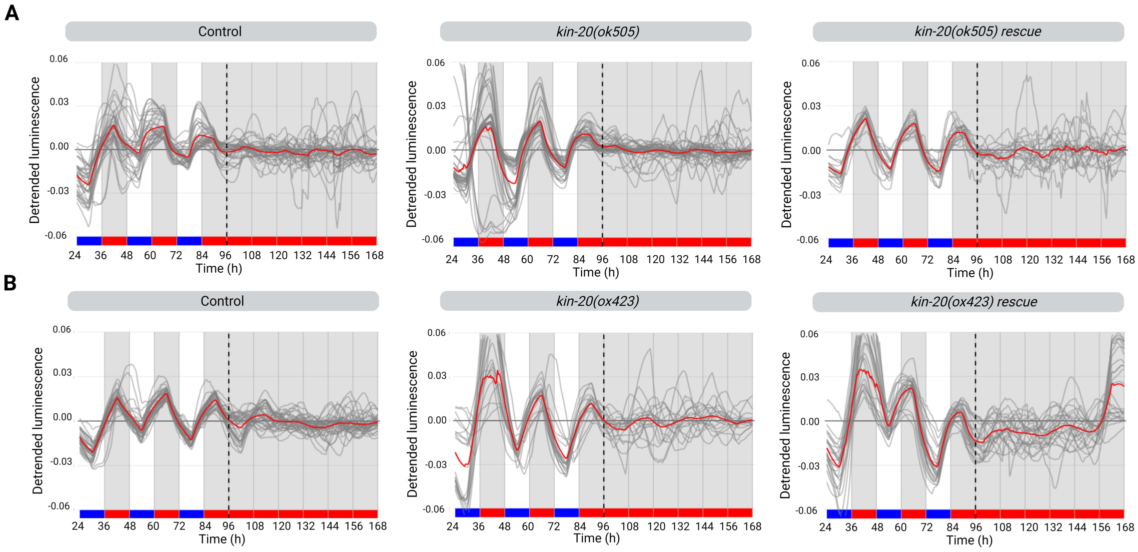

Figure S6. *kin-20* mutants are rhythmic under cyclic and FR conditions. Representative luciferase activity rhythms of adult populations under dual cyclic conditions (LD/CW, ~150/0 µmol/m^2/s; 15.5°C/17°C) and FR conditions (DD, 17°C): control (**A,** n=37), *lin-42(ox461)* (**B**, n=32), *lin-42b* overexpression (OE) transgene strain (**C**, n=20). Luminescence signals are shown as mean ± SEM in red line and all the individual wells are represented in the gray line. Each population consisted of 50 adult nematodes per well. The analysis includes three biological replicates for each strain.

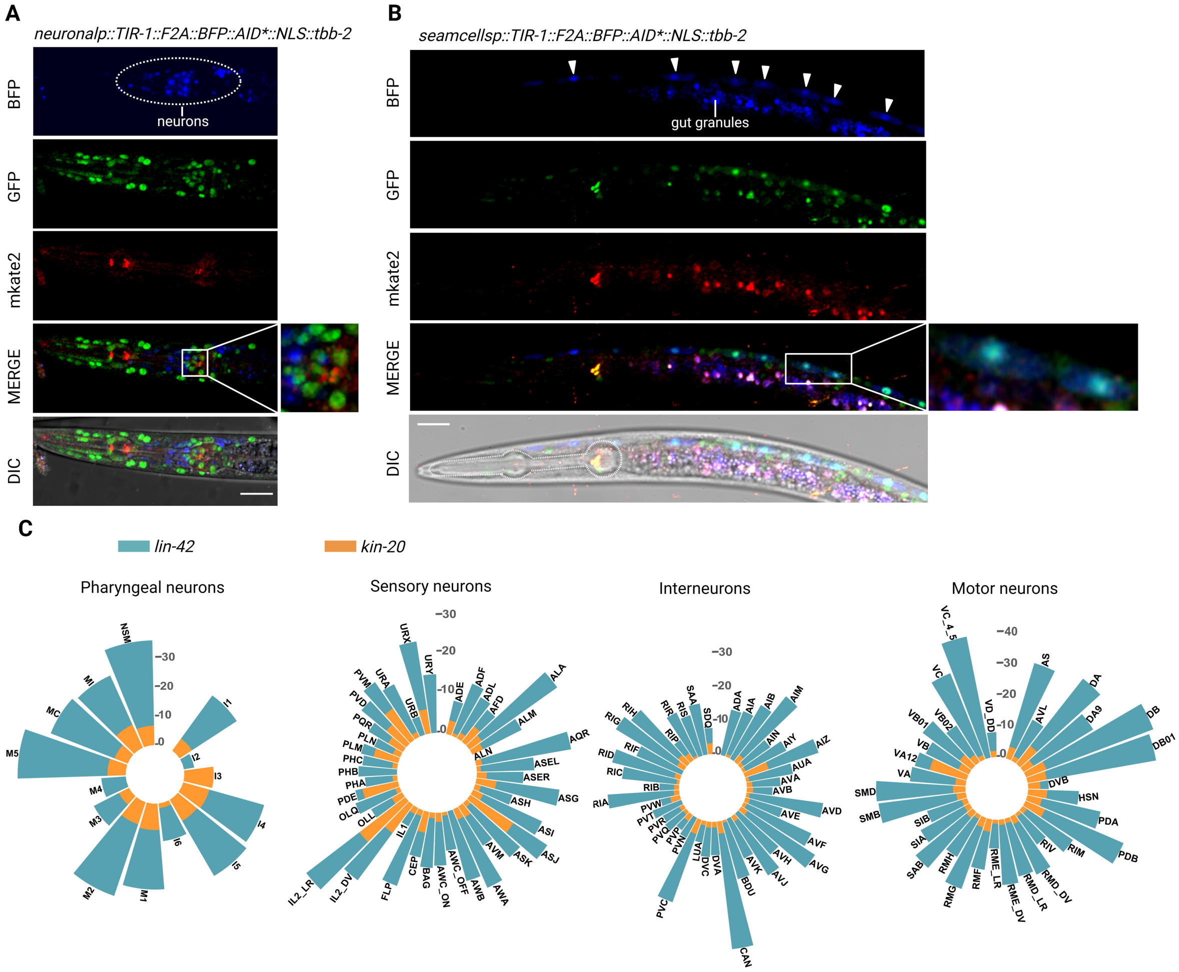

Figure S7. Co-expression of LIN-42B and KIN-20B in neurons and seam cells. A. LIN-42B::GFP (green channel) and KIN-20B::mKate2 (red channel) are detected in the neurons which also express BFP (blue channel, white circle) in L4 nematodes. Scale bars represent 20 µm. **B.** LIN-42B::GFP (green channel) and KIN-20B::mKate2 (red channel) are detected in the seam cells which also express BFP (blue channel, arrowheads) in L3/L4 nematodes. Scale bars represent 20 µm. **C.** Representative expression of *lin-42* (green bars) and *kin-20* (orange bars) in pharyngeal neurons, motor neurons, sensory neurons, and interneurons. Data obtained from CeNGEN (https://www.cengen.org/). The scale represents the percentage of expression of the genes of interest. Each bar represents a neuron.

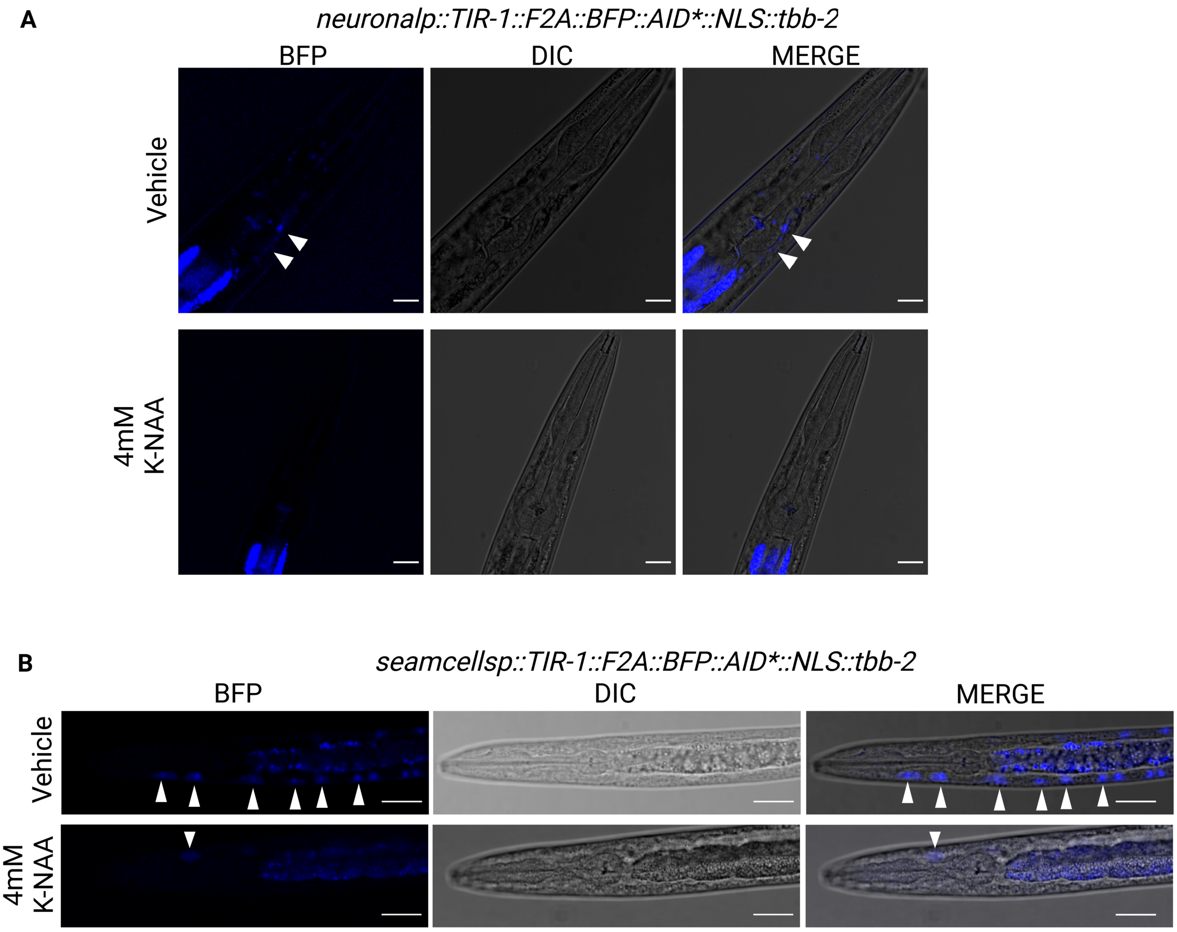

Figure S8. Expression of BFP in neurons and seam cells exposed to Auxin. Representative images of nematodes expressing the neuronal rgef-1p::TIR-1::F2A::BFP::AID*::NLS::tbb-2, (**A**) and the seam cell SCMp::TIR-1::F2A::BFP::AID*::NLS::tbb-2 reporters (**B**), treated with vehicle and 4 mM K-NAA for 7 days. An overlay of DIC and BFP images was used to show the expression of BFP-positive neuronal cells in animals at stage L3/L4 (arrowheads). Scale bars represent 20 µm.

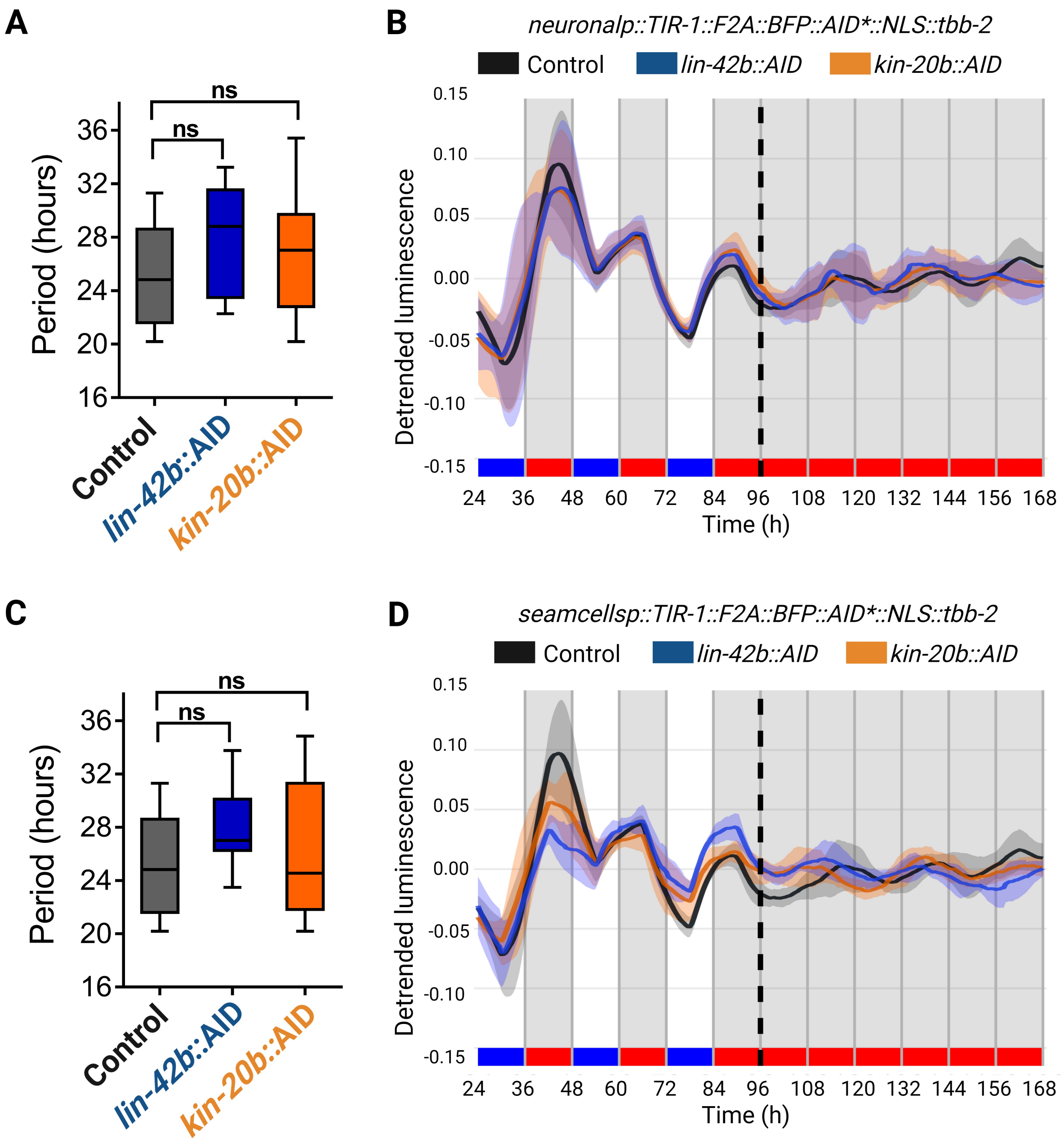

Figure S9. A. Average endogenous period of control (25.09 ± 0.68 h, n=29), *lin-42b*::AID mutants (27.62 ± 1.14 h, n=13) and *kin-20b*::AID mutants (26.93 ± 1.30 h, n=12), which also express the construct *rgef-1p*::TIR-1::F2A::BFP::AID*::NLS::tbb-2. One-way ANOVA, Dunnett's multiple comparisons test, ns. **B.** Representative luciferase activity rhythms of adult populations shown in **A**, under dual cyclic conditions (LD/CW, ~150/0 µmol/m2/s; 15.5°C/17°C) and FR conditions (DD, 17°C). **C.** Average endogenous period of control (25.09 ± 0.68 h, n=29), *lin-42b*::AID mutants (27.87 ± 0.74 h, n=14) and *kin-20b*::AID mutants (26.16 ± 1.12 h, n=17), which also express the construct *SMCp*::TIR-1::F2A::BFP::AID*::NLS::tbb-2. One-way ANOVA followed by Dunnett's multiple comparisons test, ns. **D.** Representative luciferase activity rhythms of adult populations

shown in C, under the same dual cyclic conditions and FR conditions. Each population consisted of 50 adult nematodes per well. The analysis includes three biological replicates for each strain.

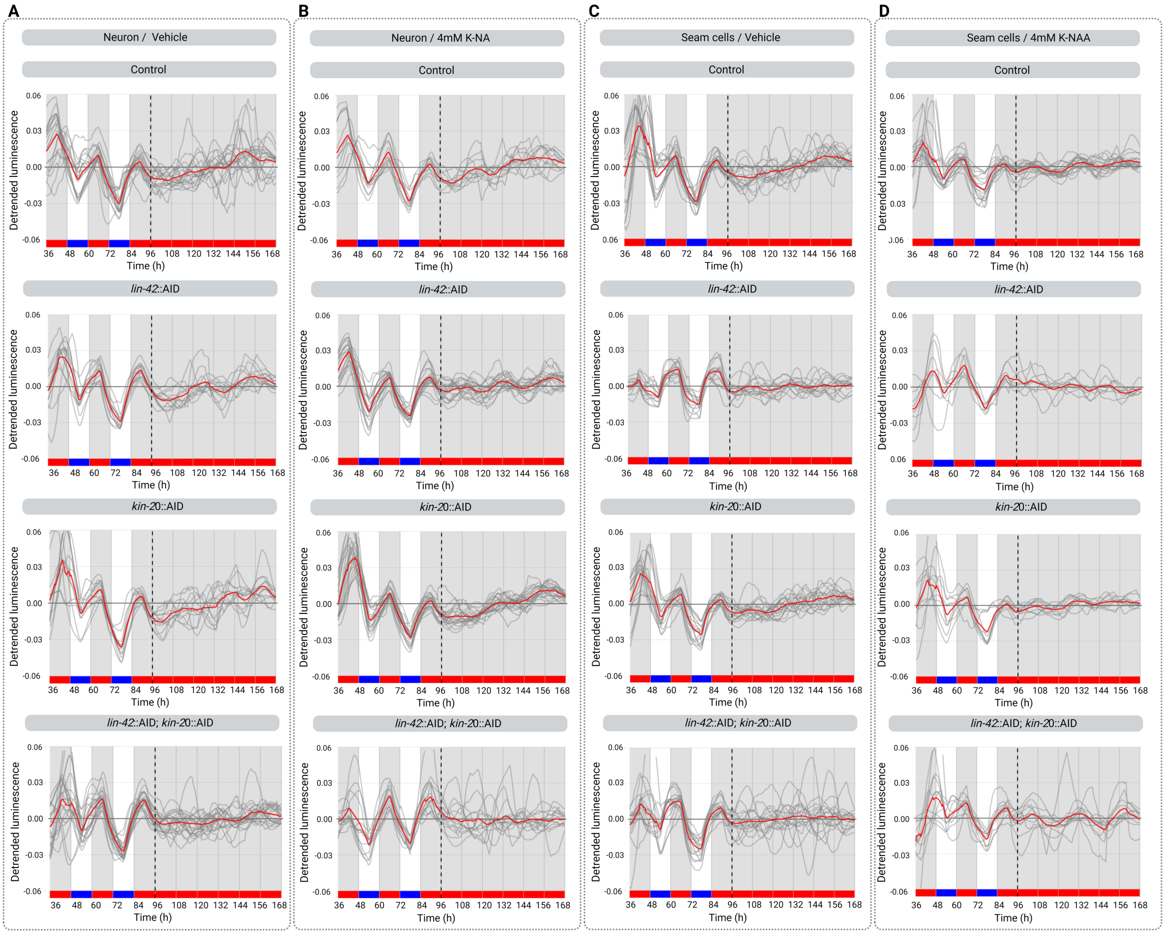

Figure S10. *lin-42*::AID and *kin-20*::AID are rhythmic under cyclic and FR conditions. Representative luciferase activity rhythms of adult populations under dual cyclic conditions (LD/CW, ~150/0 μmol/m^2/s; 15.5°C/17°C) and FR conditions (DD, 17°C) in the control strains, *lin-42*::AID, *kin-20*::AID, *lin-42*::AID;*kin-20*::AID, with vehicle (A/C) and auxin (B/D), in neuronal cells (A-B) and in seam cells (C-D). Luminescence signals are shown as mean ± SEM in red line and all the individual wells are represented in the gray line. Each population consisted of 50 adult nematodes per well. The analysis includes three biological replicates for each strain.

Data Collection and Refinement Statistics:

	**Lin42**
**Data collection**	
Beam line	APS (23IDD)
Space group	I 2 2 2
Unit cell dimensions (a,b,c) (Å), (α, β, γ (º)	50.73, 105.9, 152.9, 90 90 90
Resolution Range (Å) (highest shell)*	45.94 - 2.42 (2.51 - 2.42)
Wavelength (Å)	1.033
Total observations	114830(11082)
Unique reflections	16123 (1634)
Completeness (%)	99.52 (98.48)
R_{merge}	14(65.8)
<I/σ>	10.2(3.3)
CC1/2	99.4(89.5)
Redundancy (highest shell)	7.1(6.8)
**Refinement**	
R_{work} %/ R_{free} %	26.5 (29.5)
Number of non-hydrogen atoms	1394
Protein Residues	169
Ligand/ion	-
Water	76
B-factor (Wilson)	32.4
RMSD Bond length (Å)	0.008
RMSD Bond angle	1.25
Ramachandran favored(%)/Ramachandran outliers (%)	97.0/0.0

* Values in parenthesis are for higher resolution shell.

Supplementary Table 1. PDB

Strain	Genotype	Description
VQ1310	qvIs8(psur-5::luc::gfp + punc-122::rfp) #5OC	Luminescent rhythmic reporter in background N2
RG1590	lin-42(ox461) II	PAS and SYQ/LT domains deletion
MT2257	lin-42(n1089) II	PAS domain deletion
RB1843	lin-42(ok2385) II	SYQ/LT domain deletion
VQ1354	lin-42(ox461) II #5OC; qvIs8	ox461 mutant with the luminescent rhythmic reporter
VQ1326	lin-42(n1089) II #4OC; qvIs8	n1089 mutant with the luminescent rhythmic reporter
VQ1353	lin-42(ok2385) II #2OC; qvIs8	ok2385 mutant with the luminescent rhythmic reporter
VQ1484	lin-42(ox461) II #5OC; qvIs8; qvEx400(plin-42b/c::isoform b::gfp::unc-54; pBB107)	ox461 rescue with lin-42b and the luminescent rhythmic reporter
VC398	kin-20(ok505) X	kinase domain deletion
VQ1355	kin-20(ok505) X #5OC; qvIs8	ok505 mutant with the luminescent rhythmic reporter
VQ1539	kin-20(ok505) X #3OC; qvIs8; qvEx390(pkin-20::isoform b::rfp; pBB107)	ok505 rescue with kin-20b and the luminescent rhythmic reporter
EG5202	oxIs12(punc-47::GFP, lin-15(+)) kin-20(ox423) X	nonsense mutation (Q344stop) in kinase domain
DG1	oxIs12(punc-47::GFP, lin-15(+)) kin-20(ox423) X; qvIs8	ok423 mutant with the luminescent rhythmic reporter
EG9581	oxSi1087(pkin-20::RFP::kin-20::kin-20UTR, Cb unc-119(+) ∗ttTi5605) II ; oxIs12(punc-47::GFP, lin-15(+)) kin-20(ox423) X	ox423 rescue with kin-20a/c/d
DG2	oxSi1087(pkin-20::rfp::kin-20::kin-20UTR, Cb unc-119(+) ∗ttTi5605) II; oxIs12(punc-47::GFP, lin-15(+)) kin-20(ox423) X; qvIs8	ox423 rescue with kin-20a/c/d with the luminescent rhythmic reporter
JDW136	lin-42(wrd35 [lin-42::GFP^degron::3xFLAG]) II	native lin-42 tagged for auxin degradation (AID) and 3XFLAG
VQ1850	kin-20(syb4198[kin-20b/c::mkate-2^degron::HA]) X	native kin-20 tagged for auxin degradation (AID) and HA
JDW233	wrdSi46[SCMp*::TIR1::F2A::BFP::AID*::NLS::tbb-2 3'UTR] I:-5.32	Seam cell-specific expression of TIR1 co-factor for AID, and tissue-specific AID-tagged blue protein in seam cell nuclei. Some expression in hypodermal cells
VQ1873	wrdSi46[SCMp*::TIR1::F2A::BFP::AID*::NLS::tbb-2 3'UTR] I; qvIs8	Expression of TIR, cofactor for AID system, in seam cells, with luminescent rhythmic reporter (control)
VQ1871	lin-42(wrd35[lin-42::GFP^degron::3xFLAG]) II; wrdSi46[SCMp*::TIR1::F2A::BFP::AID*::NLS::tbb-2 3'UTR] I:-5.32; qvIs8	LIN-42 degradation in seam cells, with luminescent rhythmic reporter
VQ1872	kin-20(syb4198[kin-20b/c::mkate-2^degron::HA])X; wrdSi46[SCMp*::TIR1::F2A::BFP::AID*::NLS::tbb-2 3'UTR] I:-5.32; qvIs8	KIN-20 degradation in seam cells, with luminescent rhythmic reporter
VQ1870	kin-20(syb4198[kin-20b/c::mkate-2^degron::HA]) X; lin-42(wrd35[lin-42::GFP^degron:3xFLAG]) II; wrdSi46[SCMp*::TIR1::F2A::BFP::AID*::NLS::tbb-2 3'UTR] I:-5.32; qvIs8	LIN-42 and KIN-20 degradation in seam cells, with luminescent rhythmic reporter
DV3805	eSi7[rgef1p::TIR1::F2A::mTagBFP2::NLS::AID::tbb-2 3'UTR] (I:-5.32)	Neuronal-specific expression of TIR1 co-factor for AID, and tissue-specific AID-tagged blue protein in neuronal nuclei
VQ1903	qvIs8 (psur-5::luc::gfp + punc-122::RFP); reSi7 [rgef1p::TIR1::F2A::mTagBFP2::NLS::AID::tbb-2 3'UTR] I	Expression of TIR, cofactor for AID system, in neurons, with luminescent rhythmic reporter (control)
VQ1901	lin-42(wrd35[lin-42::GFP^degron::3xFLAG])II; reSi7 [rgef-1p::TIR1::F2A::mTagBFP2::NLS::AID::tbb-2 3'UTR] (I:-5.32); qvIs8	LIN-42 degradation in neurons, with luminescent rhythmic reporter
VQ1872	kin-20(syb4198[kin-20b/c::mkate-2^degron::HA]) X; wrdSi46[SCMp*::TIR1::F2A::BFP::AID*::NLS::tbb-2 3'UTR] I:-5.32; qvIs8	KIN-20 degradation in neurons, with luminescent rhythmic reporter
VQ1919	kin-20(syb4198[kin-20b/c::mkate-2^degron::HA]) X; lin-42(wrd35[lin-42::GFP^degron::3xFLAG])II; reSi7 [rgef-1p::TIR1::F2A::mTagBFP2::NLS::AID::tbb-2 3'UTR] (I:-5.32); qvIs8	LIN-42 and KIN-20 degradation in neurons, with luminescent rhythmic reporter
DG14	kin-20(syb4198[kin-20b/c::mkate-2^degron::HA]) X; lin-42(wrd35[lin-42::GFP^degron::3xFLAG])II; reSi7 [rgef-1p::TIR1::F2A::mTagBFP2::NLS::AID::tbb-2 3'UTR] (I:-5.32)	LIN-42::GFP and KIN-20::mKate2 expression with the marker for neuronal cells with BFP
DG15	kin-20(syb4198[kin-20b/c::mkate-2^degron::HA]) X; lin-42(wrd35[lin-42::GFP^degron::3xFLAG])II; wrdSi46[SCMp*::TIR1::F2A::BFP::AID*::NLS::tbb-2 3'UTR] (I:-5.32)	LIN-42::GFP and KIN-20::mKate2 expression with the marker for seam cells with BFP

Supplementary Table 2. Mutant strains used for the experiments

Primer Name	Sequence
KIN-20 b Fw1	CCACCGCACCGCTGGTTTCTTCAAGAAAGCGGAGATGG
KIN-20 b Fw2	TGACAACAACCCACCTCTCGGATCCATCAACCGTCAGCCACCGCACCGCTGGTTTCTTC
KIN-20 b Fw3 XbaI	GCTCTAGAACCATCCATCTGACAACAACCCACCTCTCG
KIN-20 b Rv SacI	CGAGCTCGATAATGTCGACGTTGATTTG
KIN-20 b Fw	ATTTTTGCCACGTTTTAGCTCTAGAACCATCCATCTGAC
KIN-20 b Rv	TGGCGACCGGTACCCGAGCTCGATAATGTCGACGTTGATTTG

Supplementary Table 3. Primers
